# Take Care: development and evaluation of a serious game about violence against children and adolescents*

**DOI:** 10.1590/1518-8345.7939.4737

**Published:** 2026-01-19

**Authors:** Izabela Andréa da Silva, Isabela Makiolka Montingelli, Carlos Nascimento Silla, Deborah Ribeiro Carvalho, Marcia Regina Cubas

**Affiliations:** 1 Pontifícia Universidade Católica do Paraná, Curitiba, PR, Brazil. Pontifícia Universidade Católica do Paraná PR Curitiba Brazil; 2 Halmstad University, Academy of Information Technology, Halmstad, Halland, Sweden. Halmstad University Academy of Information Technology Halland Halmstad Sweden; 3 Universidade Federal do Paraná, Curitiba, PR, Brazil. Universidade Federal do Paraná PR Curitiba Brazil

**Keywords:** Educational Technology, Violence, Child, Adolescent, Continuing Education, Play and Playthings

## Abstract

to develop a serious game for nurses and physicians in Primary Health Care based on clinical cases that address types of violence against children and adolescents, and evaluate the serious game based on player satisfaction*.*

applied research of technological nature. Four stages were carried out: development of fictitious clinical cases as a basis for the serious game; evaluation of clinical cases by eight experts intentionally selected for participating in the Protection Network; development of the serious game by an interdisciplinary team; and evaluation of satisfaction with playing the game, using the e-GameFlow scale, with the participation of 33 nurses and 15 physicians.

seventeen clinical cases were developed, representing different types of violence. “Take Care” was developed as a simulation using Unity® software, set in a virtual doctor’s office. The game’s evaluation achieved a Cronbach’s alpha of 0.970. The areas of greatest satisfaction were: improvement in knowledge, concentration, and clarity of objectives. All participants said they would play again, 91% felt safe, 89% would not give up, and 70% finished without reporting the violence.

“Take Care” was evaluated positively. It is suggested that it be used for continuing education, as an aid in identifying and reporting situations of violence.

## Highlights

**(1)** Preparation of clinical cases involving different types of violence. **(2)** Development of technology for continuing education in health. **(3)** Realistic and playful technology for continuing education in health on complex topics. **(4)** Professional improvement of reasoning and clinical skills. **(5)** Raising awareness among professionals about mandatory reporting.

## Introduction

Technology permeates the healthcare field in countless ways, including playful and interactive educational strategies through digital games, known as serious games, with specific content to achieve idealized and programmed objectives^(1)^.

A bibliometric study that analyzed 475 articles indexed in international databases reported an increase in publications on serious games applied to health education since 2018, peaking in 2023^(2)^, which determines the relevance of the topic. In contrast to passive learning methodologies, the use of serious games in health education provides interactive and immersive experiences, stimulating critical thinking, decision-making, and clinical reasoning^(3)^.

Due to their authentic, realistic, and safe environment, combined with playful features, serious games are an effective technology for learning, simulation, and training in risky or critical situations^(3-4)^. They are considered technological tools capable of modifying the process of developing competencies and skills^(4)^, as well as bringing students closer to the reality in which the knowledge will be applied, reinforcing their relevance in continuing health education (CHE).

CHE aims to improve knowledge and skills based on the demands of the profession and the experiences faced in everyday professional life^(5)^. In Brazil, its practice is consolidated in the Unified Health System (SUS) through the National Policy for Continuing Health Education (NPCHE).

In the SUS, Primary Health Care (PHC) is configured as a space dedicated to comprehensive care, promoting collaborative team practices, using different technologies for the comprehensive care of users^(5)^. Among the issues faced in PHC, violence stands out as a complex and dynamic biopsychosocial phenomenon, originating and perpetuated in the social context, which affects health, causing physical damage and mental disorders^(6)^. About 2.5% of global deaths are associated with various types of violence, and thousands of people face non-fatal assaults every day. In Brazil, children and adolescents are impacted by the problem of violence, which requires the qualification of health professionals and services to address it^(7)^.

It is up to professionals to protect children and adolescents from violence. One form of protection is through early identification and mandatory reporting of suspected or confirmed cases, both in public and private services^(7)^. However, even with legal mechanisms aimed at protecting and guaranteeing rights, the literature highlights the underreporting of violence and identifies structural weaknesses and barriers to its enforcement, including the difficulty for professionals to identify the signs and types of violence early on^(7)^. To minimize the problem, the NPCHE guidelines related to the incorporation of technologies for CHE in health services reinforce that a more interactive and dynamic approach allows for improving the qualification of health professionals^(4)^.

Based on the above and assuming that a serious game is an educational technology that aids in the identification of cases of violence, aimed at the CHE of nurses and physicians, this study had the following objectives: to develop a serious game for nurses and physicians in Primary Health Care based on clinical cases that address types of violence against children and adolescents and to evaluate the serious game based on player satisfaction.

## Method

### Study design and sample

This is an applied research project of technological nature, focused on the development of a serious game, carried out in four stages: (i) development of fictitious clinical cases as an empirical basis for the game; (ii) evaluation of the clinical cases developed; (iii) development of the game; (v) evaluation of the game.

For the stage of developing the fictitious clinical cases, the researcher—a nurse with experience in care and teaching, in the care of children and adolescents—used a previously planned script, according to an instructional model for the development of clinical cases^(8)^. This script covered the types of violence most and least reported in the Notifiable Diseases Information System (SINAN, its acronym in Portuguese). The theoretical construction of the cases was based on a review of the literature on violence against children and adolescents^(9)^, the Protocol of the Network for the Protection of Children and Adolescents at Risk of Violence in the Municipality of Curitiba, capital of the State of Paraná (PR)^(10)^, with an emphasis on instructions for completing the notification form, and on a study that used cases and investigated the difficulties faced by pediatricians in identifying and reporting child abuse^(11)^.

During the preparation of the fictitious clinical cases representing types of violence, the material underwent four rounds of preliminary analysis, conducted by a research group composed of health professionals: six doctoral students, five master’s students, five scientific initiation scholarship recipients, and two professors with PhDs. Each clinical case was structured in six parts, according to an instructional model^(8)^: (i) identification of the child or adolescent (name and age); (ii) collection of clinical data (medical history); (iii) summary of problems (relevant clinical signs); (iv) theoretical basis (technical-scientific literature); (v) diagnosis (identification of the type of violence) and (vi) references.

A qualitative approach was adopted to evaluate the clinical cases^(12)^ and verify the adequacy of the clinical case with the reality identified in the experience of the Protection Network. The selection of experts was carried out intentionally^(13)^, beginning with contacts established during meetings of the State Council for the Rights of Children and Adolescents (CEDCA, its acronym in Portuguese).

Based on the recommendation of a representative from the Protection Network for People in Situations of Violence of the Municipal Health Secretariat of Curitiba (SMS), professionals from different areas with recognized experience in the subject were selected. In addition, practical experience related to violence and academic qualifications were considered essential parameters. Due to the intentional nature of the selection, there were no exclusion criteria. Eight professionals from different backgrounds and fields participated in this stage.

The development stage of the simulation-based serious game took place in three stages: (i) pre-production – planning process; (ii) production – analysis, design, implementation, integration, and testing processes; and (iii) post-production – execution and evaluation of results^(14)^.

It should be noted that, prior to the pre-production stage, meetings were held with the multidisciplinary team to collectively understand the real problem, addressing the characteristics of the target audience and the scenario, the motivation and objective of CHE, and the skills to be improved. Health professionals and digital game specialists, including programmers, game designers, and artists, came together for the serious game development process in a collaborative and multidisciplinary manner.

For the serious game evaluation stage, an intentional selection of participants was made using the snowball technique, initiated by the recommendation of the expert from the first stage. The following inclusion criteria were established: nurses or physicians working in PHC in direct care for children or adolescents, with a minimum of two years of clinical experience. Due to the intentional nature of the selection, there were no exclusion criteria. Forty-eight experts were selected, including 15 physicians and 33 nurses, working in the ten health districts of Curitiba-PR.

### Place of study

The research was conducted in the municipality of Curitiba, capital of the state of Paraná. The municipality’s PHC network consists of 109 health centers, distributed across ten health districts. The first author of this article conducted individual face-to-face meetings with all participants at their workplaces, which were scheduled in advance and at their convenience.

### Data collection

Data collection for the clinical case evaluation stage was carried out between October 2023 and January 2024. Each expert analyzed one complete case used in the game tutorial and two cases related to types of violence, selected from among the most and least frequent in the municipality under study. All cases were analyzed by at least one judge.

The serious game evaluation data collection took place between March and July 2024. Each expert nurse and physician answered questions from a script consisting of: (i) professional characteristics; (ii) affinity with technologies; (iii) experiences with continuing education in health. The expert then played the game on a mobile device specifically used for the research and completed the eGameFlow scale.

Data collection for the evaluation of clinical cases and the serious game was carried out in a single face-to-face meeting with each of the experts, totaling 56 meetings.

### Assessment tool

Due to its descriptive nature, no specific assessment tool was used in the clinical case evaluation stage.

For the serious game evaluation stage, the eGameFlow scale^(15)^ was used, which is specific to educational games, with domains for evaluating player satisfaction, including: concentration, challenges, autonomy, clarity of objectives, feedback, immersion, and knowledge improvement. The social interaction domain was excluded because it is an individual game. The Likert scale was used, which involves assessing the level of agreement and disagreement with something, based on the player’s perception of a satisfaction scale: totally satisfied (5), very satisfied (4), satisfied (3), partially dissatisfied (2), and totally dissatisfied (1). The criterion was used for all domains and their respective items.

In addition, three questions were added at the end of the scale: (1) Would you play this game again? (2) Were there times when you thought about giving up the game? (3) Did you feel unsure about how to proceed? The answers were dichotomous, with the options “yes” or “no.”

### Data processing and analysis

The experts’ responses were organized into tables and their contributions were incorporated into the clinical cases in a descriptive manner.

Cronbach’s alpha reliability test was used to analyze the eGameFlow scale. For the analysis of gameplay, a spreadsheet was created in Excel® software, based on the game database hosted on a server. The normality of the data distribution was verified by the Shapiro-Wilk test. The criterion for determining significance was set at 5%. The statistical analysis was processed using SPSS statistical software version 22.0.

### Ethical considerations

The research was approved by the Research Ethics Committees of the Pontifical Catholic University of Paraná (PUCPR) on June 21, 2023, under Opinion No. 6,134,914 and Certificate of Presentation for Ethical Review (CAAE) No. 70498023. 2.0000.0020, and by the Municipal Health Secretariat of Curitiba on October 17, 2023, under Opinion No. 6,431,147 and CAAE No. 70498023.2.3001.0101. All participants signed the Free and Informed Consent Form before accessing the data collection instruments.

## Results

The results of the study are organized according to the four stages of the method.

In the case development stage, 17 clinical cases involving different types of violence were developed. The fictitious identification of the child or adolescent, age, and description of the types of violence focused on in the case are presented in Figure 1.

The profile of the experts who evaluated the clinical cases revealed an interdisciplinary composition, comprising three nurses, two educators, two psychologists, and one pediatrician. Regarding postgraduate education, four judges had master’s degrees, three had doctorates, and one was a specialist. Their professional experience ranged from five to 26 years.

Figure 1 describes the judges’ agreement with the types of violence and the additions to the types of violence suggested by the experts. The experts identified the presence of types of violence distinct from or additional to those initially described in cases 1, 10, 11, and 14. All additions were included in the cases.

**Figure 1-:** Description of clinical case items and judges’ evaluation. Curitiba, PR, Brazil, 2024

**Case**	**Fictitious identification**	**Age (in years)**	**Types of violence**	**Agreement with types of violence**	**Additions**
1	A.B.	Nine	Psychological violence Physical violence	Yes	Self-inflicted violence
2	M.P.	Three	Structural neglect Physical violence	Yes	-
3	T.S.	One	Physical violence Neglect of protection	Yes	-
4	R.R.	Six	Physical violence Neglect of protection	Yes	-
5	T.C.	Ten	Physical violence	Yes	-
6	Y.C.	Eleven	Psychological violence Physical violence	Yes	-
7	I.L.	Six	Neglect Psychological abuse	Yes	-
8	O.S.	Seven	Neglect of protection Psychological violence Physical violence	Yes	-
9	P.V.	Ten	Neglect of health, structural and protection Child labor	Yes	-
10	A.R.	Eight	Physical violence Psychological violence (suspected bullying at school) Neglect of protection	Yes	Psychological violence (parental alienation)
11	S.G.	Eleven	Self-inflicted or self-induced violence	Yes	Sexual violence
12	D.V.	Fifteen	Self-inflicted violence Neglect of protection	Yes	-
13	B.G.	Fifteen	Child labor Neglect of protection, health, and schooling	Yes	-
14	J.C.	Eight	Sexual violence Neglect of protection	Yes	Psychological violence
15	L.P.	Seven	Self-inflicted violence Psychological violence Neglect of protection and health	Yes	-
16	V.L.	Twelve	Child labor Structural and protective neglect	Yes	-
17	B.G.	Eight	Sexual violence Neglect of protection	Yes	-

For the serious game development stage, the simulation genre was established using Unity® software, with a 2D environment perspective, compatible with Android, iOS, and browser (itch.io) platforms.

Although the game is not characterized as a traditional clinical simulation in a face-to-face environment, its design was guided by the principles established in the Healthcare Simulation Standards of Best Practice® of the International Nursing Association for Clinical Simulation and Learning (INACSL). The game structure integrates, in a form adapted to the digital environment, the three essential stages of simulation: prebriefing, simulation, and debriefing.

The prebriefing stage was operationalized through an introductory tutorial clinical case, which guides the player through the environment, objectives, and features of the game. The player assumes the role of a nurse or doctor responsible for the clinical care of children and adolescents in a primary care setting.

In this scenario, dialog boxes with specific guidelines are presented. This initial support aims to ensure understanding of how the system works and to promote the gradual acquisition of autonomy in subsequent consultations. Next, the game presents basic patient information, such as name, age, date of consultation, and identification of the accompanying person. This information contributes to the contextualization of the case and the beginning of clinical reasoning.

The interaction intensifies with the appearance of a dialog box associated with a stethoscope icon. From that moment on, the player observes the interaction between the professional and the patient or companion, represented by images. The icon visible on the interface is updated according to the character in the scene.

The simulation stage consisted of 16 fictitious clinical cases, in which, during the interactions, consultations, medical histories, physical examinations, and clinical data are recorded in the medical record, and decisions are made based on the protocols of the Child and Adolescent Protection Network for at-risk individuals. These actions add dynamism to the scenario and enable informed clinical decisions.

Among the available options are: flagging the case as suspected violence, maintaining this flagging, or reporting suspected violence or the actual act.

The debriefing stage occurs through automated feedback throughout the game play, highlighting successes and gaps related to mandatory reporting.

The game was titled “Take Care”. The choice was justified by the English term’s literal meaning of “to take care of something or someone”. “Take Care” is in the process of being registered as software.

**Figure 2-:**
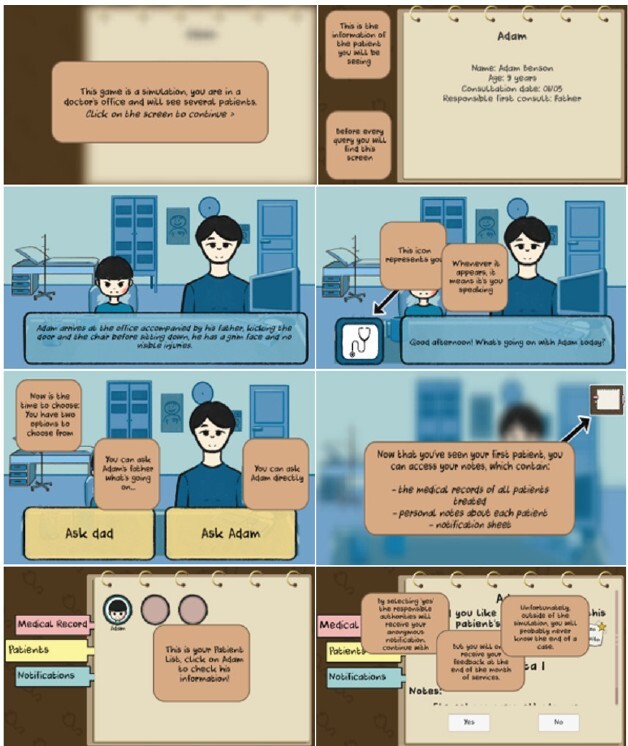
Example of screens from the tutorial for the serious game “Take Care”. Curitiba, PR, Brazil, 2024

Figure 2 shows examples of screens from the tutorial of the serious game developed.

The 48 experts who evaluated the serious game were predominantly aged between 40 and 49 (29%), followed by those aged between 30 and 39 (28%). In terms of academic qualifications, 48% had postgraduate degrees, with only one having a *stricto sensu* master’s degree. In terms of experience, 50% of them had more than 21 years of experience, and only 18% of participants had between two and five years of experience.

All experts reported an affinity with technology, and 75% responded that they had no contact with digital games.

Regarding experience with continuing education, 37.5% reported having participated in CHE less than a year ago; 54% between one and 10 years ago; and 4.5% more than 10 years ago. Of the total number of experts, 33% stated that they had not had contact with the topic of violence against children and adolescents in CHE.

As for the approaches used for CHE on the topic of violence, the most cited model was the lecture, with eight participants, followed by case discussions, with seven participants. Other models included: short courses (five participants), roundtable discussions (two participants), and lectures (three participants).

Regarding the gameplay results, the minimum execution time was between 13 and 15 minutes, while the maximum time recorded was between 21 and 30 minutes. During the simulated experience, 41% of participants identified the cases presented as suspected violence, and 29% made the mandatory notification.

The statistical analysis using Cronbach’s alpha test was 0.968. Based on standardized values, Cronbach’s alpha was 0.970, demonstrating almost perfect consistency. Table 1 shows the mean and standard deviation of each response in all domains evaluated.

**Table 1-:** Mean and standard deviation of items on the e-GameFlow scale. Curitiba, PR, Brazil, 2024

**Questions**	**Mean (n=48)**	**Standard deviation**
C*		
C1 - Does the game hold my attention?	4.20	0.889
C2 - Does it present content that stimulates my attention?	4.27	0.811
C3 - Are most activities related to the learning task?	4.33	0.801
C4 - Are there any distractions from the task?	4.00	0.957
C5 - Overall, am I able to stay focused on the game?	4.39	0.786
C6 - Am I distracted from tasks I should be focusing on?	3.18	1.185
C7 - Am I overwhelmed with tasks that seem unimportant?	3.20	1.207
C8 - Is the workload of the game appropriate?	3.98	0.989
H ^†^		
H1 - Do I enjoy the game without getting bored or anxious?	4.18	0.950
H2 - Is the difficulty appropriate?	3.90	0.941
H3 - Are there “tips” that help with the task?	4.02	1.010
H4 - Does it offer online support that helps with the task?	3.76	1.128
H5 - Does it offer video or audio that helps with the task?	3.69	1.211
H6 - Do my skills improve as the game progresses?	4.14	0.866
H7 - Am I motivated by the improvement of my skills?	4.12	1.053
H8 - Do the challenges increase as my skills improve?	3.94	0.922
H9 - Does it present new challenges at an appropriate pace?	4.00	0.890
H10 - Does it offer different levels of challenges that adapt to different players?	3.71	1.061
A ^‡^		
A1 - Do I feel in control of the menu?	3.24	1.164
A2 - Do I feel in control of functions and objects?	3.37	1.035
A3 - Do I feel in control of the interactions between functions and objects?	3.45	0.959
A4 - Is it possible to make mistakes that prevent the game from progressing?	3.04	0.999
A5 - Can I recover from any mistakes I make?	2.69	1.103
A6 - Do I feel that I can use any strategies?	3.04	0.978
A7 - Do I feel in control and that I have an impact on the game?	3.57	1.021
A8 - Do I know the next step in the game?	3.51	1.023
A9 - Do I feel in control of the game?	3.37	1.035
G ^§^		
G1 - Are the overall objectives presented at the beginning of the game?	4.20	0.957
G2 - Are the overall objectives presented clearly?	4.20	0.979
G3 - Are the intermediate objectives presented in the appropriate place?	4.06	0.944
G4 - Are the intermediate objectives presented clearly?	4.00	0.979
G5 - Do I understand the learning objectives through the game?	4.22	0.963
F||		
F1 - Do I receive feedback on my progress in the game?	4.08	1.017
F2 - Do I receive immediate feedback on my actions?	3.78	1.177
F3 - Am I notified of new tasks immediately?	4.02	0.968
F4 - Am I notified of new events immediately?	3.98	1.010
F5 - Do I receive information about the success or failure of intermediate objectives immediately?	3.51	1.120
F6 - Do I receive information about my status, such as level or score?	3.31	1.342
I ^¶^		
I1 - Do I lose track of time while playing?	3.88	1.092
I2 - Do I forget about things around me while playing?	3.88	1.033
I3 - Do I forget about everyday problems while playing?	3.76	1.011
I4 - Do I feel a sense of altered time?	3.37	0.951
I5 - Can I get involved with the game?	4.04	0.999
I6 - Do I feel emotionally involved with the game?	3.88	0.992
I7 - Do I feel viscerally involved with the game?	3.35	1.217
K**		
K1 - Does the game improve my knowledge?	4.29	0.913
K2 - Do I grasp the basic ideas of the content presented?	4.43	0.791
K3 - Do I try to apply the knowledge in the game?	4.39	0.812
K4 - Does the game motivate the player to integrate the content presented?	4.33	0.899
K5 - Do I want to know more about the content presented?	4.33	0.922

*C = Concentration; ^†^H = Challenge; ^‡^A = Autonomy; ^§^G = Clarity of objectives; ^||^F = Feedback; ^¶^I = Immersion; **K = Knowledge improvement

The results obtained for the eGameFlow scale indicate that the average of the items evaluated was 3.831, with values ranging from 2.694 (minimum) to 4.429 (maximum), resulting in a range of 1.735 and a ratio between the maximum and minimum values of 1.644. The variance of the item averages was 0.177. Regarding the variance of the items, an average of 1.016 was observed, with values ranging from 0.617 to 1.800, corresponding to a range of 1.183 and a ratio between the maximum and minimum values of 2.916. The variance of these values was 0.061.

All participants said they would play again, 92% felt safe playing, and 89% did not think about quitting the game.

## Discussion

In developing the cases, two or more different types of violence perpetrated against the same child or adolescent were used. Even so, the experts identified yet another type of violence in some cases. This overlap intensifies the impacts and is in line with research by authors who point out that one type of violence is not predominantly identified in isolation^(16)^.

During the preparation of the cases, it was considered that physical and sexual violence are the most recognized by health professionals and that they have difficulty recognizing other types of violence. Therefore, when designing the cases for CHE simulation, the overlap of recognized types of violence and types that are more difficult to identify because they do not present explicit signs was an important strategy.

Regarding the experts selected in the first stage of the research, the diversity of professional training contributed to a broader scope of cases, as it identified types of violence that allowed for overlap in clinical cases. This result is in line with the difficulty of identification and complexity of the cases evaluated in the Health Care Networks^(15)^, which reflects on the relevance of interdisciplinarity in the construction of knowledge and practice in health.

In the game development stage, with educational technology in mind, after choosing the theme, in addition to focusing on learning, it is necessary to create an intuitive and easy-to-navigate interface to promote engagement and ensure that users can easily access content whenever they want^(17)^.

Thus, in the development of the game, the use of fictional clinical cases was essential to support it and define it in the simulation genre, which used basic gameplay mechanics, using Unity® software, created a perspective for a 2D environment, compatible with Android, iOS, and browser (itch.io) platforms.

Unity® is configured as the game engine, widely used in the creation of interactive digital applications, with support for two-dimensional (2D) environments. Its architecture allows projects to run on different operating systems and devices, such as smartphones.

Unity® was used due to its intuitive graphical interface and extensive technical documentation base, factors that favor both beginners and experienced developers in building interactive and gamified experiences^(18)^.

Developed based on the principles of clinical simulation^(19)^, “Take Care” offers players the opportunity to take on the role of a nurse or doctor in primary care, in the context of caring for children and adolescents. During the simulation, players interact with different user profiles and clinical situations, being challenged to exercise clinical reasoning and sensitivity to raise hypotheses of violence.

This interactive dynamic immerses the player in a realistic scenario, in which the identification of risk situations is based on careful clinical assessment, interpretation of nonverbal language, analysis of behavioral signs, and possible inconsistencies in reports. Subtle elements such as small physical signs, evidence of neglect, and psychosocial indicators, all of which are described in the literature, are also considered^(16)^.

The development of skills to identify situations of sexual abuse and seek help was the result identified in an educational game whose objective and name reflect the concept of creating a virtual environment in which children aged 8 to 10 explore and interact safely^(17)^. Similarly, although with a different theme and target audience, “Take Care” was developed in a virtual environment with realistic scenarios and care situations, in which doctors and nurses can explore and interact with sensitive content safely. This proposal aims to promote the development of clinical reasoning, professional sensitivity, and the ability to identify and report situations of violence against children and adolescents.

This concept dialogues with the potential of serious games to simulate real clinical situations, providing users with immersive experiences that promote active learning and the improvement of clinical skills.

An example is the Game Virtual ER, a virtual emergency room focused on interprofessional education for medical and nursing students^(19)^. This tool uses a digital platform to simulate emergency room care, promoting teamwork and clinical decision-making in a safe and controlled environment^(19)^.

Complementarily, the game “Take Care” presents an innovative proposal by transposing clinical simulation to nurses and doctors in the PHC context, with a specific focus on identifying and reporting violence against children and adolescents.

By placing the player in realistic scenarios based on fictional clinical cases, “Take Care” can broaden the scope of action of health professionals, stimulating clinical reasoning for the recognition of subjective signs of suffering, which are often not identified in healthcare practice.

Although not prevalently identified in the results, it is expected that the use of “Take Care” will stimulate understanding of the importance of reporting cases of violence and coordinating with the Protection Network mechanisms, promoting integration between clinical skills, ethical sensitivity, and intersectoral action.

The predominance of nurses in the evaluation of the serious game reflects the presence of this professional in PHC care. This point is highlighted in a study that analyzed the profile and practices of PHC nurses in southern Brazil, which showed the central role of nurses in providing direct care to the population, reinforcing their predominance over other health professionals^(19)^. However, for the evaluation of the game, the participation of physicians was also important, considering that they provide direct care to children and adolescents in PHC and are professionals who identify situations of violence during consultations.

Another point to be highlighted is the professional maturity of the experts included in the evaluation of “Take Care”. Maturity was also reported in a Brazilian study, which suggested a profile of experts with a consolidated career and accumulated experience in health care^(20)^. Such a profile may favor a more careful evaluation determined by the different generational stages.

There is no consensus on the use of criteria for selecting experts to evaluate educational technologies, but there is agreement on the need for aspects related to academic training and professional performance/experience^(21)^. Thus, it is suggested that in future studies, one of the selection criteria for evaluating educational technologies for healthcare should be based on professional maturity.

The results regarding the experts’ proximity to technologies reflect, in part, the fact that society has undergone a significant social transformation, profoundly changing the ways of thinking, communicating, relating, and working. These changes are driven by the digital revolution^(22)^.

It was expected that experts would demonstrate an affinity for the use of technologies, given the growing use of digital resources in healthcare. However, the data from this study indicated that this affinity did not extend to digital games. The lack of dissemination among healthcare professionals about serious games and their educational applications may have limited prior access to this experience.

Despite this, the lack of familiarity did not compromise interaction with the serious game or understanding of the proposed content. This reinforces the inclusive potential of technology, even among professionals with different levels of exposure to digital gaming resources.

It is also important to highlight the scenario of low regularity or access to CHE. Such a scenario may suggest fragility in the institutionalization of CHE in health services and indicate gaps in updating, which compromise the quality of care. In turn, it is necessary to analyze with caution, as the effectiveness of CHE is often limited by factors such as work overload, lack of investment, and the presence of a biomedical model that favors quantitative care goals and indicators^(23)^.

Regarding game play, it is first necessary to highlight that the game time was predominantly between 13 and 15 minutes. The time may have reflected adequate planning, in which “Take Care” was designed to maintain attention and achieve objectives without overwhelming players.

During gameplay, the decision to mark a case as suspected or confirmed violence is attributed to the professional’s clinical decision. Understanding that notification is an obligation of all health professionals, constituting a crucial step in activating the intersectoral protection network^(24)^, it is concerning that players did not indicate notification at the end of the case.

These findings are consistent with national and international literature^(25)^, which points to difficulties faced by health professionals in identifying situations of violence against children and adolescents. Such gaps involve not only the identification of signs, especially the most subtle or invisible ones, but also decision-making regarding mandatory reporting^(24)^.

Regarding the evaluation of the game using the eGameFlow scale, the results indicated satisfactory performance in most of the domains analyzed. The results of the “Knowledge improvement” domain suggest that “Take Care” favored understanding, integration, and interest in the content, reinforcing its potential as a tool to support CHE.

Similarly, the domains “Concentration” and “Clarity of objectives” also showed positive results, with emphasis on items such as “I can stay focused on the game” and “I understand the learning objectives”. These findings suggest that the game design was successful in maintaining the player’s focus and presenting goals clearly, promoting cognitive engagement during the interactive simulation.

Research on the analysis and evaluation of serious games similar to the results presented in this article can be found in the literature. Research that analyzed serious games in the health field for the design and evaluation of serious games aimed at health education, involving health professionals, patients, and the general public, described that 58% of the games aimed to improve knowledge and 41% focused on skill development.

Another study on the use of serious games that evaluated the technology and effectiveness of educational action in CHE showed an improvement in knowledge after the strategy^(26)^. In both cases, the evaluation of the serious game was positive in the domain of knowledge improvement, with an average above 4.00 in all items evaluated, a result corroborated by the results of the present study, which suggests the educational purpose of a serious game.

Participants showed positive receptivity to “Take Care” when they stated that they would play again, felt safe while playing, and would not give up. This suggests that the game allowed for an immersive experience.

A study that developed a serious game in which the target audience was children and evaluated it with the same tool used in the present research presented similar results in relation to the autonomy category. In the study, it was decided to adjust the interface in the final version of the game, indicating the next item and allowing the player to prepare adequately for the next stage^(17)^. This strategy will be used for adjustments in “Take Care”.

This study contributes to the advancement of scientific knowledge by presenting an innovative proposal for educational technology aimed at identifying and reporting violence against children and adolescents in PHC. The serious game “Take Care” has demonstrated potential to strengthen public protection policies, serving as a tool to help reduce the invisibility of this phenomenon.

In addition, “Take Care” is a promising strategy for comprehensive and humanized care practices, as it promotes awareness among professionals of invisible signs and symptoms of types of violence that they would not otherwise report. “Take Care” can also be used in health courses.

Among the limitations of the research are: (i) the time required to develop the game, which is lengthy and requires adjustments that are sometimes not appropriate for the time frame of the *stricto sensu* academic program; (ii) the difficulty in maintaining the continuous engagement of the interdisciplinary teams involved in the different stages of the project; and (iii) the research scenario taking place in a capital city with a consolidated history of action in the Protection Network, which may limit the generality of the results and require adjustments to the tool or the inclusion of new clinical cases with distinct characteristics.

## Conclusion

The concepts underlying the types of violence, the guidelines of the Protocol for the Protection of Children and Adolescents at Risk, and the use of an instructional script were appropriate and essential for the development of clinical cases that represent real-life phenomena.

The serious game “Take Care” was developed by an interdisciplinary team in the simulation genre, using Unity® software, in a 2D environment, compatible with Android, iOS, and browser (itch.io) platforms, allowing for easy access.

The evaluation of “Take Care” reached a Cronbach’s alpha of 0.970. The game performed satisfactorily in the satisfaction evaluation, with an average of 3.831 for the items evaluated (minimum of 2.694 and maximum of 4.429), with a range of 1.735 and a ratio between the maximum and minimum values of 1.644. The domains with the highest satisfaction were “Improvement of knowledge”, “Concentration”, and “Clarity of objectives”.

It is hoped that the “Take Care” serious game will help nurses and doctors in primary care to identify and report situations of violence, recognizing the phenomenon as a complex and multifaceted expression rooted in social relations.

## Data Availability

The dataset of this article is available on the Doctoral dissertation “Casos clínicos e jogo sério para a educação permanente em saúde para identificação de violência a criança e adolescente” at the link https://pergamum-biblioteca.pucpr.br/acervo/375395
