# Supplementary material for: Take Care: development and evaluation of a serious game about violence against children and adolescents
Source: Rev Lat Am Enfermagem. 2026 Jan 19;34:e4737. doi: 10.1590/1518-8345.7939.4737 (PMC12854654; doi:10.1590/1518-8345.7939.4737)
Supplement: Translation

# Translation: *Take Care*: desenvolvimento e avaliação jogo sério sobre violência contra criança e adolescente*

**DOI:** 10.1590/1518-8345.7939.4738

**Authors:** Izabela Andréa da Silva, Isabela Makiolka Montingelli, Carlos Nascimento Silla, Deborah Ribeiro Carvalho, Marcia Regina Cubas

**Affiliations:** 1 Pontifícia Universidade Católica do Paraná, Curitiba, PR, Brasil. Pontifícia Universidade Católica do Paraná PR Curitiba Brasil; 2 Halmstad University, Academy of Information Technology, Halmstad, Halland, Suécia. Halmstad University Academy of Information Technology Halland Halmstad Suécia; 3 Universidade Federal do Paraná, Curitiba, PR, Brasil. Universidade Federal do Paraná PR Curitiba Brasil

**Keywords:** Tecnologia Educacional, Violência, Criança, Adolescente, Educação Continuada, Jogos e Brinquedos

## Abstract

desenvolver um *serious game* para enfermeiros e médicos da Atenção Primária à Saúde a partir de casos clínicos que abordam tipologias de violência contra crianças e adolescentes e avaliar o *serious game*, pela satisfação do jogador*.*

pesquisa aplicada, de natureza tecnológica. Foram realizadas quatro etapas: elaboração de casos clínicos fictícios como base para o *serious game*; avaliação dos casos clínicos, por oito *experts* selecionados intencionalmente por participarem de Rede de Proteção; desenvolvimento do *serious game* por equipe interdisciplinar; e avaliação da satisfação em jogar, por meio da aplicação da escala *e-GameFlow*, com participação de 33 enfermeiros e 15 médicos.

foram elaborados 17 casos clínicos, representando diferentes tipos de violência. O *Take Care* foi desenvolvido no gênero de simulação, no *softwareUnity*®, ambientado em consultório virtual. A avaliação do jogo atingiu alfa de Cronbach 0,970. Os domínios de melhor satisfação foram: melhoria do conhecimento, concentração e clareza dos objetivos. Todos os participantes afirmaram que jogariam novamente, 91% se sentiram seguros, 89% não desistiriam e 70% finalizaram sem notificar a violência.

o *Take Care* foi avaliado positivamente. Sugere-se que ele seja usado para a educação permanente, como auxílio na identificação e notificação de situações de violência.

## Destaques

**(1)** Elaboração de casos clínicos com distintos tipos de violência. **(2)** Desenvolvimento de tecnologia para educação permanente em saúde. **(3)** Tecnologia realística e lúdica para a educação permanente em saúde de temas complexos. **(4)** Aprimoramento profissional do raciocínio e das habilidades clínicas. **(5)** Sensibilização de profissionais para a notificação obrigatória.

## Introdução

A tecnologia permeia a área da saúde em inúmeras possibilidades, dentre elas a estratégia educativa lúdica e interativa por meio de jogos digitais, conhecida como *serious game*, com conteúdo específico para atingir objetivos idealizados e programados^(1)^.

O estudo bibliométrico que analisou 475 artigos indexados em bases internacionais relatou um crescimento nas publicações sobre *serious games* aplicados à educação em saúde, a partir de 2018, com pico em 2023^(2)^, o que determina a atualidade do tema. Em contraste com metodologias de aprendizagem passivas, o uso de *serious games* na educação em saúde proporciona experiências interativas e imersivas, estimulando o pensamento crítico, a tomada de decisão e o raciocínio clínico^(3)^.

Devido aos seus aspectos de ambiente autêntico, realista e seguro, aliados a recursos lúdicos, os *serious games* configuram-se como uma tecnologia eficaz para a aprendizagem, simulação e treinamento em situações de risco ou críticas^(3-4)^. Eles são considerados ferramentas tecnológicas capazes de modificar o processo de desenvolvimento de competências e habilidades^(4)^, bem como para realizar uma aproximação com a realidade em que o conhecimento será aplicado, reforçando sua relevância na educação permanente em saúde (EPS).

A EPS visa aprimorar os conhecimentos e habilidades a partir das demandas da atuação e das experiências enfrentadas no cotidiano profissional^(5)^. No Brasil, sua prática é consolidada no Sistema Único de Saúde (SUS), por meio da Política Nacional de Educação Permanente em Saúde (PNEPS).

No SUS, a Atenção Primária à Saúde (APS) configura-se como um espaço voltado ao cuidado integral, promovendo práticas colaborativas em equipe, utilizando diferentes tecnologias para o cuidado integral dos usuários^(5)^. Entre as questões enfrentadas na APS, destaca-se a violência, um fenômeno biopsicossocial complexo e dinâmico, originado e perpetuado no contexto social, que afeta a saúde, gerando danos físicos e transtornos mentais^(6)^. Cerca de 2,5% das mortes globais estão associadas aos diversos tipos de violência e, diariamente, milhares de pessoas enfrentam agressões não fatais. No Brasil, crianças e adolescentes são impactados pela problemática da violência, o que exige a qualificação dos profissionais e dos serviços de saúde para seu enfrentamento^(7)^.

Cabe aos profissionais protegerem as crianças e os adolescentes contra a violência. Umas das formas de proteção se dá pela identificação precoce e notificação obrigatória de casos suspeitos ou confirmados, tanto nos serviços públicos quanto nos privados^(7)^. Entretanto, mesmo com aparatos legais que visam proteger e garantir os direitos, a literatura evidencia a subnotificação da violência e identifica fragilidades estruturais e barreiras para efetivá-la, dentre elas, cita-se a dificuldade do profissional para identificar precocemente os sinais e tipos de violência^(7)^. Para minimizar o problema, as diretrizes da PNEPS relacionadas à incorporação de tecnologias para a EPS nos serviços de saúde reforçam que uma abordagem mais interativa e dinâmica permite melhorar a qualificação dos profissionais de saúde^(4)^.

Com base no exposto e tendo como pressuposto que um *serious game* é uma tecnologia educativa de auxílio para identificação de casos de violência, voltada à EPS de enfermeiros e médicos, este estudo teve como objetivos: desenvolver um *serious game* para enfermeiros e médicos da Atenção Primária à Saúde a partir de casos clínicos que abordam tipologias de violência contra crianças e adolescentes e avaliar o *serious game*, pela satisfação do jogador*.*

## Método

### Delineamento do estudo e amostra

Trata-se de uma pesquisa aplicada, de natureza tecnológica, voltada ao desenvolvimento de um *serious game*, elaborada em quatro etapas: (i) elaboração de casos clínicos fictícios como base empírica para o jogo; (ii) avaliação dos casos clínicos elaborados; (iii) desenvolvimento do jogo; (v) avaliação do jogo.

Para a etapa de elaboração dos casos clínicos fictícios, a pesquisadora – enfermeira com experiência assistencial e docente, no atendimento a crianças e adolescentes – utilizou um roteiro previamente planejado, conforme modelo instrucional para elaboração de casos clínicos^(8)^. O roteiro contemplou os tipos de violência mais e menos notificados no Sistema de Informação de Agravos de Notificação (SINAN). A construção teórica dos casos foi fundamentada em revisão de literatura sobre violência contra crianças e adolescentes^(9)^, no Protocolo da Rede de Proteção à Criança e ao Adolescente em Situação de Risco para a Violência do Município de Curitiba, capital do Estado do Paraná (PR)^(10)^, com ênfase no instrutivo para preenchimento da ficha de notificação, e em estudo que usou casos e investigou as dificuldades enfrentadas por médicos pediatras na identificação e notificação de maus-tratos na infância^(11)^.

Durante a elaboração dos casos clínicos fictícios que representaram tipologias de violência, o material foi submetido a quatro rodadas de análise preliminar, conduzidas em grupo de pesquisa composto por profissionais da saúde: seis doutorandos, cinco mestrandos, cinco bolsistas de iniciação científica e duas docentes doutoras. Cada caso clínico foi estruturado em seis partes, conforme modelo instrucional^(8)^: (i) identificação da criança ou adolescente (nome e idade); (ii) coleta de dados clínicos (anamnese); (iii) resumo dos problemas (sinais clínicos relevantes); (iv) fundamentação teórica (literatura técnico-científica); (v) diagnóstico (identificação do tipo de violência) e (vi) referências.

Para a avaliação dos casos clínicos, adotou-se uma abordagem qualitativa^(12)^, para verificar a adequação do caso clínico com a realidade identificada na vivência da Rede de Proteção. A seleção dos *experts* foi realizada intencionalmente^(13)^, iniciando-se com contatos estabelecidos durante reuniões do Conselho Estadual dos Direitos da Criança e do Adolescente (CEDCA).

A partir de indicação de um representante da Rede de Proteção às Pessoas em Situação de Violência da Secretaria Municipal de Saúde de Curitiba (SMS), foram selecionados profissionais de distintas áreas com reconhecida experiência na temática. Em adição, considerou-se como parâmetros essenciais a experiência prática relacionada à violência e a titulação acadêmica. Pela natureza intencional da seleção, não houve critério de exclusão. Desta etapa, participaram oito profissionais de diferentes formações e campos de atuação.

A etapa de desenvolvimento *do serious game,* no gênero de simulação, se deu em três fases: (i) pré-produção – processo de planejamento; (ii) produção – processos de análise, projeto, implementação, integração e teste; e (iii) pós-produção – processos de execução e avaliação dos resultados^(14)^.

Ressalta-se que, antes da fase de pré-produção, foram realizadas reuniões com a equipe multidisciplinar para compreensão coletiva do problema real, abordando características do público-alvo e do cenário, a motivação e o objetivo da EPS e as habilidades a serem aprimoradas. Reuniram-se para o processo de desenvolvimento do *serious game*, de forma colaborativa e multidisciplinar, profissionais da saúde e especialistas em jogos digitais, sendo programador, *game designer* e artista.

Para a etapa de avaliação do *serious game*, realizou-se uma seleção intencional de participantes com uso da técnica de bola de neve, iniciada pela indicação do *expert* da primeira etapa. Foram estabelecidos os seguintes critérios de inclusão: enfermeiro ou médico atuante na APS no atendimento direto à criança ou adolescente, com o mínimo de dois anos de experiência clínica. Pela natureza intencional da seleção, não houve critério de exclusão. Foram selecionados 48 *experts*, sendo 15 médicos e 33 enfermeiros, atuantes nos dez distritos sanitários de Curitiba-PR.

### Local de estudo

A pesquisa foi realizada no Município de Curitiba, capital do Estado do Paraná. A rede APS do município é composta por 109 Unidades de Saúde, distribuídas em dez distritos sanitários. A primeira autora deste artigo conduziu encontros presenciais individuais com todos os participantes em seus locais de trabalho, previamente agendados e respeitando sua conveniência.

### Coleta de dados

A coleta referente à etapa de avaliação dos casos clínicos foi realizada entre outubro de 2023 e janeiro de 2024. Cada *expert* analisou um caso completo utilizado no tutorial do jogo e dois casos relacionados aos tipos de violência, selecionados entre os mais e menos frequentes no município de estudo. Todos os casos foram analisados por, no mínimo, um juiz.

A coleta de avaliação do *serious game* ocorreu entre março e julho de 2024. Cada enfermeiro e médico *expert* respondeu perguntas de um roteiro composto por: (i) características profissionais; (ii) afinidade com tecnologias; (iii) experiências com educação permanente em saúde. Em seguida, o *expert* realizou o *gameplay*, por meio de um dispositivo *mobile* com uso específico para a pesquisa, e preencheu a escala *eGameFlow*.

A coleta de dados referente à avaliação dos casos clínicos e à avaliação do *serious game* foi realizada em único encontro presencial com cada um dos *experts*, totalizando 56 encontros.

### Instrumento de avaliação

Pela característica descritiva, na etapa de avaliação dos casos clínicos não foi utilizado instrumento próprio de avaliação.

Para a etapa de avaliação do *serious game*, utilizou-se a escala *eGameFlow*^(15)^, específica para jogos educacionais, com domínios de avaliação da satisfação do jogador, que incluem: concentração, desafios, autonomia, clareza dos objetivos, *feedback*, imersão e melhoria do conhecimento. O domínio interação social foi excluído por tratar-se de um jogo individual. Empregou-se a escala Likert, que envolve a avaliação do nível de concordância e discordância em relação a algo, com base na percepção do jogador quanto a uma escala de satisfação: totalmente satisfeito (5), muito satisfeito (4), satisfeito (3), parcialmente insatisfeito (2) e totalmente insatisfeito (1). O critério foi utilizado para todos os domínios e seus respectivos itens.

Complementarmente, três perguntas foram acrescentadas ao final da escala: (1) Você jogaria este jogo novamente? (2) Houve momentos em que você pensou em desistir do jogo? (3) Você se sentiu inseguro(a) sobre como proceder? As respostas eram dicotômicas, com as opções “sim” ou “não”.

### Tratamento e análise dos dados

As respostas dos *experts* foram organizadas em quadros e as contribuições foram incorporadas aos casos clínicos, de forma descritiva.

Para a análise da escala *eGameFlow*, estabeleceu-se o teste de confiabilidade coeficiente alfa de Cronbach. Para análise do *gameplay* criou-se planilha no *softwareExcel*®, a partir do banco de dados do jogo hospedado num servidor. A normalidade na distribuição dos dados foi verificada pelo teste de Shapiro-Wilk. O critério de determinação de significância adotado foi o nível de 5%. A análise estatística foi processada pelo *software* estatístico SPSS versão 22.0.

### Aspectos éticos

A pesquisa foi aprovada pelos Comitês de Ética em Pesquisa da Pontifícia Universidade Católica do Paraná (PUCPR), em 21 de junho de 2023, sob Parecer nº 6.134.914 e Certificado de Apresentação para Apreciação Ética (CAAE) nº 70498023.2.0000.0020, e da Secretaria Municipal da Saúde de Curitiba, em 17 de outubro 2023, sob Parecer nº 6.431.147 e CAAE nº 70498023.2.3001.0101. Todos os participantes assinaram o Termo de Consentimento Livre e Esclarecido antes de acessarem os instrumentos de coleta de dados.

## Resultados

Os resultados do estudo estão organizados conforme as quatro etapas do método.

Na etapa de elaboração dos casos, foram elaborados 17 casos clínicos com distintos tipos de violência. A identificação fictícia da criança ou adolescente, idade e descrição dos tipos de violência focados no caso estão apresentados na Figura 1.

O perfil dos *experts* que realizaram a avaliação dos casos clínicos revelou uma composição interdisciplinar, abrangendo três enfermeiros, dois pedagogos, dois psicólogos e um médico pediatra. Em relação ao nível de pós-graduação, quatro juízes possuíam mestrado, três, doutorado e um era especialista. O tempo de experiência profissional variou entre cinco e 26 anos.

Na Figura 1 são descritos a concordância dos juízes com os tipos de violência e os acréscimos de tipos de violência sugeridos pelos *experts*. Os *experts* identificaram a presença de tipos de violência distintos ou adicionais àqueles inicialmente descritos, nos casos 1, 10, 11 e 14. Todos os acréscimos foram acrescentados aos casos.

**Figura 1-:** Descrição de itens do caso clínico e avaliação dos juízes. Curitiba, PR, Brasil, 2024

**Caso**	**Identificação fictícia**	**Idade (em anos)**	**Tipos de violência**	**Concordância com os tipos de violência**	**Acréscimos**
1	A.B.	Nove	Violência psicológica Violência física	Sim	Violência autoprovocada
2	M.P.	Três	Negligência estrutural Violência física	Sim	-
3	T.S.	Um	Violência física Negligência de proteção	Sim	-
4	R.R.	Seis	Violência física Negligência de proteção	Sim	-
5	T.C.	Dez	Violência física	Sim	-
6	Y.C.	Onze	Violência psicológica Violência física	Sim	-
7	I.L.	Seis	Negligência Violência psicológica	Sim	-
8	O.S.	Sete	Negligência de proteção Violência psicológica Violência física	Sim	-
9	P.V.	Dez	Negligência de saúde, estrutural e de proteção Trabalho infantil	Sim	-
10	A.R.	Oito	Violência física Violência psicológica (suspeita de *bullying* na escola) Negligência de proteção	Sim	Violência psicológica (alienação parental)
11	S.G.	Onze	Violência autoprovocada ou autoinfligida	Sim	Violência sexual
12	D.V.	Quinze	Violência autoprovocada Negligência de proteção	Sim	-
13	B.G.	Quinze	Trabalho infantil Negligência de proteção, saúde e escola	Sim	-
14	J.C.	Oito	Violência sexual Negligência de proteção	Sim	Violência psicológica
15	L.P.	Sete	Violência autoprovocada Violência psicológica Negligência de proteção e saúde	Sim	-
16	V.L.	Doze	Trabalho infantil Negligência estrutural e de proteção	Sim	-
17	B.G.	Oito	Violência sexual Negligência de proteção	Sim	-

Para a etapa de desenvolvimento do *serious game*, estabeleceu-se o gênero simulação, por meio do *softwareUnity*®, em perspectiva para ambiente 2D, compatível com as plataformas *Android*, iOS e navegador (itch.io).

Embora o jogo não se caracterize como uma simulação clínica tradicional em ambiente presencial, sua concepção foi orientada pelos princípios estabelecidos nos *Healthcare Simulation Standards of Best Practice*® da *International Nursing Association for Clinical Simulation and Learning* (INACSL). A estrutura do jogo integra, de forma adaptada ao ambiente digital, as três fases essenciais da simulação: *prebriefing*, simulação e *debriefing.*

A fase de *prebriefing* foi operacionalizada por meio de um caso clínico tutorial introdutório, que orienta o jogador quanto ao ambiente, aos objetivos e às funcionalidades do jogo. O jogador assume o papel de enfermeiro ou médico responsável pelo atendimento clínico de crianças e adolescentes em contexto de APS.

Nesse cenário, são apresentadas caixas de diálogo com orientações específicas. Esse apoio inicial visa garantir a compreensão do funcionamento do sistema e favorecer a aquisição gradual de autonomia nas consultas subsequentes. Em seguida, o jogo apresenta as informações básicas do paciente, tais como nome, idade, data da consulta e identificação do acompanhante. Essas informações contribuem para a contextualização do caso e para o início do raciocínio clínico.

A interação se intensifica a partir da aparição de uma caixa de diálogo associada a um ícone de estetoscópio. A partir desse momento, o jogador observa a interação entre o profissional e o paciente ou acompanhante, representados por imagens. O ícone visível na interface é atualizado conforme o personagem em cena.

A fase de simulação foi composta por 16 casos clínicos fictícios, nos quais durante as interações, realiza-se consulta, anamnese, exame físico, registra-se dados clínicos no prontuário e toma-se decisões baseadas nos protocolos da Rede de Proteção à Criança e ao Adolescente em situação de risco. Tais ações conferem dinamismo ao cenário e possibilitam decisões clínicas fundamentadas.

Entre as opções disponíveis, estão: sinalizar o caso como suspeito de violência, manter essa sinalização ou efetuar a notificação de suspeita de violência ou do ato concretizado.

A fase de *debriefing* ocorre por meio de *feedbacks* automatizados ao longo do *gameplay*, destacando acertos e lacunas relacionados a notificação obrigatória.

O jogo foi intitulado *Take Care*. A escolha foi justificada pelo termo em inglês ter o significado literal de “cuidar de algo ou alguém”. O *Take Care* encontra-se em processo de registro de *software*.

Na Figura 2 estão exemplos de telas do tutorial do *serious game* desenvolvido.

**Figura 2-:** Exemplo das telas do tutorial do *serious gameTake Care*. Curitiba, PR, Brasil, 2024

Os 48 *experts* que realizaram a avaliação do *serious game* eram da faixa etária predominante de 40 a 49 anos (29%), seguida de 30 a 39 anos (28%). Com relação à formação acadêmica, 48% possuíam pós-graduação, sendo apenas uma *stricto sensu*, nível mestrado. Quanto ao tempo de experiência, verificou-se que 50% deles tinham mais de 21 anos de experiência e apenas 18% dos participantes tinham um tempo entre dois e cinco anos.

Todos os *experts* relataram afinidade com a tecnologia e 75% responderam que não possuíam contato com jogos digitais. Em relação à experiência com educação permanente, 37,5% informaram ter realizado a última participação na EPS havia menos de um ano; 54% entre um e 10 anos; e 4,5% mais de 10 anos. Do total de experts, 33% afirmaram que não haviam tido contato com o tema da violência contra criança e adolescente na EPS.

Quanto às abordagens utilizadas para a EPS sobre o tema da violência, o modelo mais citado foi a aula expositiva, com oito participações, seguido pela discussão de casos, com sete participações. Outros modelos incluíram: curso de pequena duração (cinco participações), roda de conversa (duas participações) e palestra (três participações).

Em relação aos resultados do *gameplay*, o tempo mínimo de execução foi entre 13 e 15 minutos, enquanto o tempo máximo registrado situou-se entre 21 e 30 minutos. Durante a experiência simulada, 41% dos participantes identificaram os casos apresentados como suspeitos de violência e 29% realizaram a notificação obrigatória.

A análise estatística pelo teste de alfa de Cronbach foi de 0,968. Com base em valores padronizados, o alfa de Cronbach foi de 0,970 – demonstrando consistência quase perfeita. A Tabela 1 apresenta a média e o desvio-padrão de cada resposta em todos os domínios avaliados.

**Tabela 1-:** Média e desvio-padrão dos itens da escala *e-GameFlow*. Curitiba, PR, Brasil, 2024

**Questões**	**Média (n=48)**	**Desvio-padrão**
C*		
C1 - O jogo prende minha atenção?	4,20	0,889
C2 - Apresenta conteúdo que estimula minha atenção?	4,27	0,811
C3 - A maioria das atividades está relacionada com a tarefa da aprendizagem?	4,33	0,801
C4 - Nenhuma distração da tarefa é destacada?	4,00	0,957
C5 - No geral, consigo ficar concentrado no jogo?	4,39	0,786
C6 - Sou distraído de tarefas nas quais deveria me concentrar?	3,18	1,185
C7- Sou sobrecarregado com tarefas que parecem sem importância?	3,20	1,207
C8 - A carga de trabalho do jogo é adequada?	3,98	0,989
H ^†^		
H1 - Aproveito o jogo sem ficar entediado ou ansioso?	4,18	0,950
H2 - A dificuldade é adequada?	3,90	0,941
H3 - Existem “dicas” que ajudam na tarefa?	4,02	1,010
H4 - Apresenta suporte *on-line* que ajuda na tarefa?	3,76	1,128
H5 - Apresenta vídeo ou áudio que ajudam na tarefa?	3,69	1,211
H6 - Minhas habilidades aumentam conforme o jogo avança?	4,14	0,866
H7 - Sou motivado pela melhora das minhas habilidades?	4,12	1,053
H8 - Os desafios aumentam conforme minhas habilidades aumentam?	3,94	0,922
H9 - Apresenta novos desafios em um ritmo adequado?	4,00	0,890
H10 - Apresenta diferentes níveis de desafios que se adaptam aos diferentes jogadores?	3,71	1,061
A ^‡^		
A1 - Tenho sensação de controle do menu?	3,24	1,164
A2 - Tenho sensação de controle sobre funções e objetos?	3,37	1,035
A3 - Tenho sensação de controle sobre as interações entre funções e objetos?	3,45	0,959
A4 - É possível cometer erros que impedem o avanço do jogo?	3,04	0,999
A5 - Posso me recuperar de qualquer erro cometido?	2,69	1,103
A6 - Sinto que posso usar quaisquer estratégias?	3,04	0,978
A7 - Tenho sensação de controle e impacto sobre o jogo?	3,57	1,021
A8 - Sei o próximo passo no jogo?	3,51	1,023
A9 - Tenho sensação de controle sobre o jogo?	3,37	1,035
G ^§^		
G1 - Os objetivos gerais são apresentados no início do jogo?	4,20	0,957
G2 - Os objetivos gerais são apresentados claramente?	4,20	0,979
G3 - Os objetivos intermediários são apresentados no local apropriado?	4,06	0,944
G4 - Os objetivos intermediários são apresentados claramente?	4,00	0,979
G5 - Eu entendo os objetivos do aprendizado através do jogo?	4,22	0,963
F *^||^*		
F1 - Recebo *feedback* do meu progresso no jogo?	4,08	1,017
F2 - Recebo *feedback* imediato das minhas ações?	3,78	1,177
F3 - Sou notificado sobre novas tarefas imediatamente?	4,02	0,968
F4 - Sou notificado sobre novos eventos imediatamente?	3,98	1,010
F5 - Recebo informação sobre sucesso ou falha de objetivos intermediários imediatamente?	3,51	1,120
F6 - Recebo informação sobre o meu *status* , como nível ou pontuação?	3,31	1,342
I ^¶^		
I1 - Esqueço do tempo enquanto jogo?	3,88	1,092
I2 - Esqueço das coisas ao meu redor enquanto jogo?	3,88	1,033
I3 - Esqueço dos problemas do dia a dia enquanto jogo?	3,76	1,011
I4 - Sinto uma noção de tempo alterada?	3,37	0,951
I5 - Posso ficar envolvido com o jogo?	4,04	0,999
I6 - Me sinto emocionalmente envolvido com o jogo?	3,88	0,992
I7 - Me sinto visceralmente envolvido com o jogo?	3,35	1,217
K ^**^		
K1 - O jogo melhora meu conhecimento?	4,29	0,913
K2 - Capto as ideias básicas do conteúdo apresentado?	4,43	0,791
K3 - Tento aplicar o conhecimento no jogo?	4,39	0,812
K4 - O jogo motiva o jogador a integrar o conteúdo apresentado?	4,33	0,899
K5 - Quero saber mais sobre o conteúdo apresentado?	4,33	0,922

*C = Concentração; †H = Desafio; ‡A = Autonomia; §G = Clareza dos objetivos; ||F = *Feedback*; ¶I = Imersão; **K = Melhoria do conhecimento

Os resultados obtidos para a escala *eGameFlow* indicam que a média dos itens avaliados foi de 3,831, com valores variando entre 2,694 (mínimo) e 4,429 (máximo), resultando em uma amplitude de 1,735 e uma razão entre o valor máximo e o mínimo de 1,644. A variância das médias dos itens foi de 0,177. No que se refere à variância dos itens, observou-se uma média de 1,016, com valores oscilando entre 0,617 e 1,800, correspondendo a uma amplitude de 1,183 e a uma razão entre o valor máximo e o mínimo de 2,916. A variância desses valores foi de 0,061.

Todos os participantes afirmaram que jogariam novamente, 92% sentiram-se seguros ao jogar e 89% não pensaram em desistir do jogo.

## Discussão

Na elaboração dos casos utilizou-se dois ou mais diferentes tipos de violência perpetrada na mesma criança ou adolescente. Ainda assim, os *experts* identificaram mais outro tipo de violência, em alguns casos. Essa sobreposição intensifica os impactos e está alinhada com a pesquisa de autores que apontam que um tipo de violência não é identificado de forma isolada, predominantemente^(16)^.

Durante a elaboração dos casos considerou-se que a violência física e sexual são as mais reconhecidas pelos profissionais de saúde e que eles possuem dificuldade para reconhecer outros tipos de violência. Portanto, conceber os casos com objetivo de simulação para EPS, a sobreposição de tipos de violência reconhecidos e tipos mais difíceis de identificar, por não apresentarem sinais explícitos^(16)^, foram estratégias importantes.

Em relação aos *experts* selecionados na primeira etapa da pesquisa, a diversidade de formação profissional contribuiu para maior abrangência dos casos, porque possibilitou identificar tipos de violência que viabilizam a sobreposição nos casos clínicos. Este resultado está alinhado com a dificuldade de identificação e complexidade dos casos avaliados nas Redes de Atenção à Saúde^(15)^, o que reflete a relevância da interdisciplinaridade na construção do conhecimento e na prática em saúde.

Na etapa de desenvolvimento do jogo, com propósito de tecnologia educacional, após a escolha do tema, além do foco no aprendizado é necessária a criação de interface intuitiva e fácil de navegar, para promover o engajamento e assegurar que os usuários consigam acessar facilmente os conteúdos sempre que desejarem^(17)^.

Assim, no desenvolvimento do jogo, o uso de casos clínicos fictícios foi essencial para sustentá-lo e para defini-lo no gênero de simulação, o qual utilizou mecânicas básicas de jogabilidade, por meio do *softwareUnity*®, criando perspectiva para ambiente 2D, compatível com as plataformas *Android*, iOS e navegador (itch.io).

O *Unity*® configura-se como o motor de jogo, amplamente empregado na criação de aplicações interativas digitais, com suporte a ambientes bidimensionais (2D). Sua arquitetura permite que os projetos sejam executados em diferentes sistemas operacionais e dispositivos, como *smartphones*.

O *Unity*® foi utilizado devido a sua interface gráfica intuitiva e uma extensa base de documentação técnica, fatores que favorecem tanto iniciantes quanto desenvolvedores experientes na construção de experiências interativas e gamificadas^(18)^.

Desenvolvido com base nos princípios da simulação clínica^(19)^, o *Take Care* oferece ao jogador a oportunidade de assumir o papel de enfermeiro ou médico da APS, no contexto de atendimento a crianças e adolescentes. Durante a simulação, o jogador interage com diferentes perfis de usuários e situações clínicas, sendo desafiado a exercer raciocínio clínico e sensibilidade para levantar hipóteses de violência.

Essa dinâmica interativa promove a imersão do jogador em um cenário realista, em que a identificação de situações de risco se dá a partir da avaliação clínica cuidadosa, da interpretação da linguagem não verbal, da análise de sinais comportamentais e das possíveis inconsistências nos relatos. Também são considerados elementos sutis como pequenos sinais físicos, indícios de negligência e indicadores psicossociais, todos eles descritos em literatura^(16)^.

O desenvolvimento de habilidades para identificar situações de abuso sexual e buscar ajuda foi o resultado identificado em um jogo educacional cujo objetivo e nome refletem o conceito de criar um ambiente virtual, no qual crianças de oito a 10 anos exploram e interagem de forma segura^(17)^. Do mesmo modo, embora com temática e público-alvo distintos, o *Take Care* foi desenvolvido em ambiente virtual com cenários e atendimentos realistas, nos quais médicos e enfermeiros podem explorar e interagir com conteúdo sensível de forma segura. Essa proposta visa favorecer o desenvolvimento do raciocínio clínico, da sensibilidade profissional e da capacidade de identificar e notificar situações de violência contra crianças e adolescentes.

Essa concepção dialoga com o potencial dos *serious games* para simular situações clínicas reais, proporcionando aos usuários experiências imersivas que favorecem o aprendizado ativo e o aprimoramento de habilidades clínicas.

Um exemplo é o jogo *Virtual ER*, um pronto-socorro virtual voltado à educação interprofissional de estudantes de medicina e enfermagem^(19)^. Essa ferramenta utiliza uma plataforma digital para simular atendimentos em salas de emergência, promovendo o trabalho em equipe e a tomada de decisões clínicas em ambiente seguro e controlado^(19)^.

De forma complementar, o jogo *Take Care* apresenta uma proposta inovadora ao transpor a simulação clínica para enfermeiros e médicos no contexto APS, com foco específico na identificação e notificação da violência contra crianças e adolescentes.

Ao inserir o jogador em cenários realistas e baseados em casos clínicos fictícios, o *Take Care* pode ampliar o escopo de atuação dos profissionais da saúde, estimulando o raciocínio clínico para o reconhecimento de sinais subjetivos de sofrimento, muitas vezes não identificados na prática assistencial.

Embora não identificado de forma prevalente nos resultados, espera-se que o uso do *Take Care* estimule a compreensão sobre a importância da notificação dos casos de violência e da articulação com os mecanismos da Rede de Proteção, promovendo a integração entre habilidades clínicas, sensibilidade ética e atuação intersetorial.

Em relação à predominância de enfermeiros na avaliação do *serious game*, ela é reflexo da presença desse profissional na assistência em APS. Este ponto é destacado em um estudo que analisou o perfil e as práticas de enfermeiros da APS na região Sul do Brasil, o qual evidenciou a centralidade de enfermeiros na prestação do cuidado direto à população, reforçando sua predominância frente a outros profissionais de saúde^(19)^. Entretanto, para a avaliação do jogo, a participação de médicos foi importante, considerando que eles realizam atendimento direto a crianças e adolescentes na APS e são profissionais que identificam situações de violência durante as consultas.

Outro ponto a ser destacado é a maturidade profissional dos experts incluídos na avaliação do *Take Care*. A maturidade também foi relatada em estudo brasileiro, que sugeriu um perfil de *experts* com trajetória consolidada e experiência acumulada no cuidado em saúde^(20)^. Tal perfil pode favorecer uma avaliação mais criteriosa determinada pelas distintas fases geracionais.

O uso de critérios para seleção de *experts* para avaliar tecnologias educacionais não possui consenso, mas convergem para necessidade de aspectos relacionados à formação acadêmica, atuação/experiência profissional^(21)^. Assim, sugere-se que, em estudos futuros, um dos critérios de escolha para avaliação de tecnologias educacionais para o cuidado em saúde seja fundamentado pela maturidade profissional.

Os resultados referentes à proximidade dos *experts* com tecnologias refletem, em parte, o fato de que a sociedade tem passado por uma transformação social significativa, alterando profundamente as formas de pensar, comunicar, relacionar-se e trabalhar. Essas mudanças são impulsionadas pela revolução digital^(22)^.

Era esperado que os *experts* demonstrassem afinidade com o uso de tecnologias, dado o crescente uso de recursos digitais na saúde. No entanto, os dados deste estudo indicaram que essa afinidade não se estendeu aos jogos digitais. A escassa divulgação entre profissionais da saúde sobre *serious games* e suas aplicações educativas pode ter limitado o acesso prévio a essa experiência.

Apesar disso, a ausência de familiaridade não comprometeu a interação com o *serious game* ou a compreensão dos conteúdos propostos. Isso reforça o potencial inclusivo da tecnologia, mesmo entre profissionais com diferentes níveis de exposição a recursos lúdico-digitais.

Também é importante destacar o cenário de baixa regularidade ou de acesso à EPS. Tal cenário pode sugerir fragilidade da institucionalização da EPS nos serviços de saúde e indicar lacunas de atualização, que comprometem a qualificação do cuidado. Por sua vez, é necessário analisar com parcimônia, pois a efetividade da EPS é frequentemente limitada por fatores como a sobrecarga de trabalho, falta de investimento e presença de modelo biomédico que privilegia metas e indicadores quantitativos de assistência^(23)^.

Quanto ao *gameplay*, primeiramente é necessário destacar que o tempo de jogo foi efetivado, predominantemente, entre 13 e 15 minutos. O tempo pode ter sido reflexo do planejamento adequado, em que o *Take Care* foi projetado para manter a atenção e atingir os objetivos sem sobrecarregar os jogadores.

No transcorrer do *gameplay*, a decisão de marcar um caso como suspeito ou a confirmação de violência é atribuída à decisão clínica do profissional. Compreendendo que a notificação é uma obrigação de todos os profissionais de saúde, constituindo-se como etapa crucial para o acionamento da rede intersetorial de proteção^(24)^, é preocupante o achado de que jogadores não indicaram a notificação ao final do caso_._

Esses achados dialogam com a literatura nacional e internacional^(25)^, que apontam dificuldades de profissionais da saúde para a identificação de situações de violência contra crianças e adolescentes. Tais lacunas envolvem não apenas a identificação dos sinais, especialmente os mais sutis ou invisíveis, mas também a tomada de decisão frente à notificação obrigatória^(24)^.

Quanto à avaliação do jogo por meio da escala *eGameFlow*, os resultados indicaram desempenho satisfatório na maioria dos domínios analisados. Os resultados do domínio “Melhoria do conhecimento” sugerem que o *Take Care* favoreceu a compreensão, a integração e o interesse pelo conteúdo, reforçando seu potencial como ferramenta de apoio à EPS.

De modo semelhante, os domínios “Concentração” e “Clareza dos objetivos” também apresentaram resultados positivos, com destaque para itens como “consigo ficar concentrado no jogo” e “entendo os objetivos do aprendizado”. Esses achados sugerem que o *design* do jogo foi bem-sucedido em manter o foco do jogador e apresentar metas de forma clara, favorecendo o engajamento cognitivo durante a simulação interativa.

São encontradas na literatura pesquisas sobre análise e avaliação de *serious game* semelhantes aos resultados apresentados neste artigo. A pesquisa que analisou *serious game* na área de saúde para o *design* e a avaliação de jogos sérios voltados à educação em saúde, envolvendo profissionais de saúde, pacientes e público em geral, descreveu que 58% dos jogos visavam aprimorar o conhecimento e 41% focavam no desenvolvimento de habilidades.

Outro estudo sobre o uso de *serious game* que avaliou a tecnologia e a efetividade da ação educativa na EPS evidenciou melhoria do conhecimento após a estratégia^(26)^. Em ambos, a avaliação do *serious game* foi positiva no domínio de melhoria do conhecimento, com média acima de 4,00 em todos os itens avaliados, resultado corroborado pelos resultados da presente pesquisa, o que sugere a finalidade educativa de um *serious game*.

Os participantes demonstraram receptividade positiva ao *Take Care*, quando afirmaram que jogariam novamente e sentiram-se seguros ao jogar e não desistiriam. Isso sugere que o jogo permitiu uma experiência imersiva.

Um estudo que desenvolveu *serious game* no qual o público-alvo eram crianças e que o avaliou com a mesma ferramenta usada na presente pesquisa apresentou resultados semelhantes em relação à categoria autonomia; no estudo, optou-se por ajustar a interface na versão final do jogo, indicando o item seguinte e permitindo que o jogador se preparasse adequadamente para a próxima etapa^(17)^. Essa estratégia será usada para ajustes no *Take Care*.

Este estudo contribui para o avanço do conhecimento científico ao apresentar uma proposta inovadora de tecnologia educacional voltada à identificação e notificação da violência contra crianças e adolescentes na APS. O *serious game Take Care* demonstrou potencial para fortalecer políticas públicas de proteção, sendo ferramenta para auxiliar na redução da invisibilidade desse fenômeno.

Além disso, o *Take Care* configura-se como uma estratégia promissora para práticas de cuidado integrais e humanizadas, ao promover a sensibilização dos profissionais para sinais e sintomas invisíveis de tipologias de violência, para os quais eles não tomariam decisão de notificar. De forma complementar, o *Take Care* pode ser usado em cursos da área da saúde.

Entre as limitações da pesquisa, estão: (i) o tempo exigido para o desenvolvimento do jogo, que é prolongado e exige adequações de percurso que, por vezes, não são adequadas à temporalidade do percurso acadêmico do *stricto sensu*; (ii) a dificuldade em manter o engajamento contínuo das equipes interdisciplinares envolvidas nas diferentes etapas do projeto; e (iii) o cenário de pesquisa se dar em capital com histórico consolidado de atuação em Rede de Proteção, o que pode limitar a generalidade dos resultados e necessitar de ajustes na ferramenta ou inserção de novos casos clínicos com características distintas.

## Conclusão

Os conceitos que fundamentam os tipos de violência, as diretrizes do Protocolo da Rede de Proteção à Criança e ao Adolescente em situação de risco e o uso de um roteiro instrucional foram adequados e são essenciais para a elaboração de casos clínicos que representam fenômenos da realidade.

O *serious gameTake Care* foi desenvolvido, por equipe interdisciplinar, no gênero de simulação, no *software Unity*®, em perspectiva para ambiente 2D, compatível com as plataformas *Android*, iOS e navegador (itch.io), o que lhe permite fácil acesso.

A avaliação do *Take Care* atingiu alfa de Cronbach 0,970. O jogo apresentou desempenho satisfatório na avaliação de satisfação, com média dos itens avaliados de 3,831 (mínimo de 2,694 e máximo de 4,429), com amplitude de 1,735 e razão entre o valor máximo e o mínimo de 1,644. Os domínios de melhor satisfação foram “Melhoria do conhecimento”, “Concentração” e “Clareza dos objetivos”.

Espera-se que o *serious gameTake Care* contribua com enfermeiros e médicos da APS para identificação e notificação de situações de violência, reconhecendo o fenômeno como uma expressão complexa e multifacetada, enraizado nas relações sociais.

## Data Availability

O conjunto de dados deste artigo está disponível na tese de doutorado “Casos clínicos e jogo sério para a educação permanente em saúde para identificação de violência a criança e adolescente”, no link https://pergamum-biblioteca.pucpr.br/acervo/375395.

# Translation: *Take Care*: desarrollo y evaluación de *serious game* sobre violencia contra niños y adolescentes*

**DOI:** 10.1590/1518-8345.7939.4736

**Authors:** Izabela Andréa da Silva, Isabela Makiolka Montingelli, Carlos Nascimento Silla, Deborah Ribeiro Carvalho, Marcia Regina Cubas

**Affiliations:** 1 Pontifícia Universidade Católica do Paraná, Curitiba, PR, Brasil. Pontifícia Universidade Católica do Paraná PR Curitiba Brasil; 2 Halmstad University, Academy of Information Technology, Halmstad, Halland, Suecia. Halmstad University Academy of Information Technology Halland Halmstad Suecia; 3 Universidade Federal do Paraná, Curitiba, PR, Brasil. Universidade Federal do Paraná PR Curitiba Brasil

**Keywords:** Tecnología Educacional, Violencia, Niño, Adolescente, Educación Continua, Juego e Implementos de Juego

## Abstract

desarrollar un *serious game* para enfermeros y médicos de la Atención Primaria de Salud a partir de casos clínicos que abordan tipologías de violencia contra niños y adolescentes y evaluar el *serious game* por la satisfacción del jugador.

investigación aplicada, de naturaleza tecnológica. Se realizaron cuatro etapas: elaboración de casos clínicos ficticios como base para el *serious game*; evaluación de los casos clínicos por ocho expertos seleccionados intencionalmente por participar en la Red de Protección; desarrollo del *serious game* por un equipo interdisciplinario; y evaluación de la satisfacción al jugar mediante la aplicación de la escala *e-GameFlow*, con participación de 33 enfermeros y 15 médicos

se elaboraron 17 casos clínicos que representan diferentes tipos de violencia. *Take Care* fue desarrollado en el género de simulación, en el *software Unity*®, ambientado en consultorio virtual. La evaluación del juego alcanzó alfa de Cronbach 0,970. Los dominios de mayor satisfacción fueron: mejora del conocimiento, concentración y claridad de los objetivos. Todos los participantes afirmaron que jugarían nuevamente, 91% se sintieron seguros, 89% no desistirían y 70% finalizaron sin notificar la violencia.

*Take Care* fue evaluado positivamente. Se sugiere que sea utilizado para la educación permanente, como apoyo en la identificación y notificación de situaciones de violencia.

## Destacados

**(1)** Elaboración de casos clínicos con distintos tipos de violencia. **(2)** Desarrollo de tecnología para la educación permanente en salud. **(3)** Tecnología realista y lúdica para la educación permanente en salud de temas complejos. **(4)** Perfeccionamiento profesional del razonamiento y de las habilidades clínicas. **(5)** Sensibilización de profesionales para la notificación obligatoria.

## Introducción

La tecnología permea el área de la salud en innumerables posibilidades, entre ellas la estrategia educativa lúdica e interactiva por medio de juegos digitales, conocida como *serious game*, con contenido específico para alcanzar objetivos idealizados y programados^(1)^.

Estudio bibliométrico que analizó 475 artículos indexados en bases internacionales reportó un crecimiento en las publicaciones sobre *serious games* aplicados a la educación en salud a partir de 2018, con pico en 2023^(2)^, lo que determina la actualidad del tema. En contraste con metodologías de aprendizaje pasivas, el uso de *serious games* en la educación en salud proporciona experiencias interactivas e inmersivas, estimulando el pensamiento crítico, la toma de decisiones y el razonamiento clínico^(3)^.

Debido a sus aspectos de ambiente auténtico, realista y seguro, aliados a recursos lúdicos, los *serious games* se configuran como una tecnología eficaz para el aprendizaje, simulación y entrenamiento en situaciones de riesgo o críticas^(3-4)^. Son considerados herramientas tecnológicas capaces de modificar el proceso de desarrollo de competencias y habilidades^(4)^, así como para realizar una aproximación con la realidad en que el conocimiento será aplicado, reforzando su relevancia en la educación permanente en salud (EPS).

La EPS busca perfeccionar los conocimientos y habilidades a partir de las demandas de la actuación y de las experiencias enfrentadas en el cotidiano profesional^(5)^. En Brasil, su práctica está consolidada en el Sistema Único de Salud (SUS), por medio de la Política Nacional de Educación Permanente en Salud (PNEPS).

En el SUS, la Atención Primaria de Salud (APS) se configura como un espacio orientado al cuidado integral, promoviendo prácticas colaborativas en equipo y utilizando diferentes tecnologías para el cuidado integral de los usuarios^(5)^. Entre las cuestiones enfrentadas en la APS se destaca la violencia, un fenómeno biopsicosocial complejo y dinámico, originado y perpetuado en el contexto social, que afecta la salud generando daños físicos y trastornos mentales^(6)^. Cerca del 2,5% de las muertes globales están asociadas a los diversos tipos de violencia y, diariamente, miles de personas enfrentan agresiones no fatales. En Brasil, niños y adolescentes son impactados por la problemática de la violencia, lo que exige la calificación de los profesionales y de los servicios de salud para su enfrentamiento^(7)^.

Incumbe a los profesionales proteger a los niños y adolescentes contra la violencia. Una de las formas de protección se da por la identificación precoz y la notificación obligatoria de casos sospechosos o confirmados, tanto en los servicios públicos como en los privados^(7)^. Sin embargo, incluso con aparatos legales que buscan proteger y garantizar los derechos, la literatura evidencia la subnotificación de la violencia e identifica fragilidades estructurales y barreras para efectivarla, entre ellas la dificultad del profesional para identificar precozmente las señales y tipos de violencia^(7)^. Para minimizar el problema, las directrices de la PNEPS relacionadas con la incorporación de tecnologías para la EPS en los servicios de salud refuerzan que un abordaje más interactivo y dinámico permite mejorar la calificación de los profesionales de salud^(4)^.

Con base en lo expuesto y teniendo como presupuesto que un *serious game* es una tecnología educativa de apoyo para identificación de casos de violencia, orientada a la EPS de enfermeros y médicos, este estudio tuvo como objetivos: desarrollar un *serious game* para enfermeros y médicos de la Atención Primaria de Salud a partir de casos clínicos que abordan tipologías de violencia contra niños y adolescentes y evaluar el *serious game* por la satisfacción del jugador.

## Método

### Diseño del estudio y muestra

Se trata de una investigación aplicada, de naturaleza tecnológica, orientada al desarrollo de un *serious game*, elaborada en cuatro etapas: (i) elaboración de casos clínicos ficticios como base empírica para el juego; (ii) evaluación de los casos clínicos elaborados; (iii) desarrollo del juego; (v) evaluación del juego.

Para la etapa de elaboración de los casos clínicos ficticios, la investigadora – enfermera con experiencia asistencial y docente en la atención a niños y adolescentes – utilizó un guion previamente planificado, conforme modelo instruccional para elaboración de casos clínicos^(8)^. Este guion contempló los tipos de violencia más y menos notificados en el Sistema de Información de Agravios de Notificación (SINAN). La construcción teórica de los casos fue fundamentada en revisión de literatura sobre violencia contra niños y adolescentes^(9)^, en el Protocolo de la Red de Protección al Niño y al Adolescente en Situación de Riesgo por Violencia del Municipio de Curitiba, capital del Estado de Paraná (PR)^(10)^, con énfasis en el instructivo para el llenado de la ficha de notificación, y en estudio que utilizó casos e investigó las dificultades enfrentadas por médicos pediatras en la identificación y notificación de malos tratos en la infancia^(11)^.

Durante la elaboración de los casos clínicos ficticios que representaron tipologías de violencia, el material fue sometido a cuatro rondas de análisis preliminar, conducidas en grupo de investigación compuesto por profesionales de la salud: seis doctorandos, cinco maestrandos, cinco becarios de iniciación científica y dos docentes doctoras. Cada caso clínico fue estructurado en seis partes, conforme modelo instruccional^(8)^: (i) identificación del niño o adolescente (nombre y edad); (ii) recolección de datos clínicos (anamnesis); (iii) resumen de los problemas (signos clínicos relevantes); (iv) fundamentación teórica (literatura técnico-científica); (v) diagnóstico (identificación del tipo de violencia) y (vi) referencias.

Para la evaluación de los casos clínicos se adoptó un abordaje cualitativo^(12)^, para verificar la adecuación del caso clínico con la realidad identificada en la vivencia de la Red de Protección. La selección de los expertos fue realizada intencionalmente^(13)^, iniciándose con contactos establecidos durante reuniones del Consejo Estatal de los Derechos del Niño y del Adolescente (CEDCA).

A partir de la indicación de un representante de la Red de Protección a las Personas en Situación de Violencia de la Secretaría Municipal de Salud de Curitiba (SMS), fueron seleccionados profesionales de distintas áreas con reconocida experiencia en la temática. Además, se consideró como parámetros esenciales la experiencia práctica relacionada con la violencia y la titulación académica. Por la naturaleza intencional de la selección, no hubo criterio de exclusión. De esta etapa participaron ocho profesionales de diferentes formaciones y campos de actuación.

La etapa de desarrollo del *serious game*, en el género de simulación, se dio en tres fases: (i) preproducción – proceso de planificación; (ii) producción – procesos de análisis, diseño, implementación, integración y prueba; y (iii) posproducción – procesos de ejecución y evaluación de los resultados^(14)^.

Se resalta que, antes de la fase de preproducción, se realizaron reuniones con el equipo multidisciplinario para la comprensión colectiva del problema real, abordando características del público objetivo y del escenario, la motivación y el objetivo de la EPS y las habilidades que debían perfeccionarse. Se reunieron para el proceso de desarrollo del *serious game*, de forma colaborativa y multidisciplinaria, profesionales de la salud y especialistas en juegos digitales, siendo programador, *game designer* y artista.

Para la etapa de evaluación del *serious game* se realizó una selección intencional de participantes con uso de la técnica de bola de nieve, iniciada por la indicación del experto de la primera etapa. Fueron establecidos los siguientes criterios de inclusión: enfermero o médico actuante en la APS en la atención directa al niño o adolescente, con un mínimo de dos años de experiencia clínica. Por la naturaleza intencional de la selección, no hubo criterio de exclusión. Fueron seleccionados 48 expertos, siendo 15 médicos y 33 enfermeros, actuantes en los diez distritos sanitarios de Curitiba-PR.

### Lugar del estudio

La investigación fue realizada en el Municipio de Curitiba, capital del Estado de Paraná. La red de APS del municipio está compuesta por 109 Unidades de Salud, distribuidas en diez distritos sanitarios. La primera autora de este artículo condujo encuentros presenciales individuales con todos los participantes en sus lugares de trabajo, previamente agendados y respetando su conveniencia.

### Recolección de datos

La recolección referente a la etapa de evaluación de los casos clínicos fue realizada entre octubre de 2023 y enero de 2024. Cada experto analizó un caso completo utilizado en el tutorial del juego y dos casos relacionados con los tipos de violencia, seleccionados entre los más y menos frecuentes en el municipio de estudio. Todos los casos fueron analizados por, como mínimo, un juez.

La recolección de evaluación del *serious game* ocurrió entre marzo y julio de 2024. Cada enfermero y médico experto respondió preguntas de un guion compuesto por: (i) características profesionales; (ii) afinidad con tecnologías; (iii) experiencias con educación permanente en salud. En seguida, el experto realizó el *gameplay* por medio de un dispositivo móvil con uso específico para la investigación y completó la escala *eGameFlow*.

La recolección de datos referente a la evaluación de los casos clínicos y a la evaluación del *serious game* fue realizada en un único encuentro presencial con cada uno de los expertos, totalizando 56 encuentros.

### Instrumento de evaluación

Por la característica descriptiva, en la etapa de evaluación de los casos clínicos no se utilizó instrumento propio de evaluación.

Para la etapa de evaluación del *serious game* se utilizó la escala *eGameFlow*^(15)^, específica para juegos educacionales, con dominios de evaluación de la satisfacción del jugador, que incluyen: concentración, desafíos, autonomía, claridad de los objetivos, *feedback*, inmersión y mejora del conocimiento. El dominio interacción social fue excluido por tratarse de un juego individual. Se empleó la escala Likert, que implica la evaluación del nivel de concordancia y discordancia en relación con algo, con base en la percepción del jugador en cuanto a una escala de satisfacción: totalmente satisfecho (5), muy satisfecho (4), satisfecho (3), parcialmente insatisfecho (2) y totalmente insatisfecho (1). El criterio fue utilizado para todos los dominios y sus respectivos ítems.

Complementariamente, tres preguntas fueron añadidas al final de la escala: (1) ¿Usted jugaría este juego nuevamente? (2) ¿Hubo momentos en que pensó en desistir del juego? (3) ¿Se sintió inseguro(a) sobre cómo proceder? Las respuestas eran dicotómicas, con las opciones “sí” o “no”.

### Tratamiento y análisis de los datos

Las respuestas de los expertos fueron organizadas en cuadros y las contribuciones fueron incorporadas a los casos clínicos, de forma descriptiva.

Para el análisis de la escala *eGameFlow* se estableció el coeficiente alfa de Cronbach como prueba de confiabilidad. Para el análisis del *gameplay* se creó una hoja de cálculo en el *software Excel*®, a partir de la base de datos del juego alojada en un servidor. La normalidad en la distribución de los datos fue verificada por la prueba de Shapiro-Wilk. El criterio de determinación de significancia adoptado fue el nivel del 5%. El análisis estadístico fue procesado por el *software* estadístico SPSS versión 22.0.

### Aspectos éticos

La investigación fue aprobada por los Comités de Ética en Investigación de la Pontificia Universidad Católica de Paraná (PUCPR), el 21 de junio de 2023, bajo Dictamen n.º 6.134.914 y Certificado de Presentación para Apreciación Ética (CAAE) n.º 70498023.2.0000.0020, y de la Secretaría Municipal de Salud de Curitiba, el 17 de octubre de 2023, bajo Dictamen n.º 6.431.147 y CAAE n.º 70498023.2.3001.0101. Todos los participantes firmaron el Consentimiento Informado antes de acceder a los instrumentos de recolección de datos.

## Resultados

Los resultados del estudio están organizados conforme las cuatro etapas del método.

En la etapa de elaboración de los casos se elaboraron 17 casos clínicos con distintos tipos de violencia. La identificación ficticia del niño o adolescente, edad y descripción de los tipos de violencia enfocados en el caso se presentan en la Figura 1.

El perfil de los expertos que realizaron la evaluación de los casos clínicos reveló una composición interdisciplinaria, abarcando tres enfermeros, dos pedagogos, dos psicólogos y un médico pediatra. En relación con el nivel de posgrado, cuatro jueces poseían maestría, tres doctorado y uno era especialista. El tiempo de experiencia profesional varió entre cinco y 26 años.

En la Figura 1 se describen la concordancia de los jueces con los tipos de violencia y los añadidos de tipos de violencia sugeridos por los expertos. Los expertos identificaron la presencia de tipos de violencia distintos o adicionales a los inicialmente descritos en los casos 1, 10, 11 y 14. Todos los añadidos fueron incorporados a los casos.

**Figura 1-:** Descripción de ítems del caso clínico y evaluación de los jueces. Curitiba, PR, Brasil, 2024

**Caso**	**Identificación ficticia**	**Edad (en años)**	**Tipos de violencia**	**Concordancia con los tipos de violencia**	**Añadidos**
1	A.B.	Nueve	Violencia psicológica Violencia física	Sí	Violencia autoprovocada
2	M.P.	Tres	Negligencia estructural Violencia física	Sí	-
3	T.S.	Uno	Violencia física Negligencia de protección	Sí	-
4	R.R.	Seis	Violencia física Negligencia de protección	Sí	-
5	T.C.	Diez	Violencia física	Sí	-
6	Y.C.	Once	Violencia psicológica Violencia física	Sí	-
7	I.L.	Seis	Negligencia Violencia psicológica	Sí	-
8	O.S.	Siete	Negligencia de protección Violencia psicológica Violencia física	Sí	-
9	P.V.	Diez	Negligencia de salud, estructural y de protección Trabajo infantil	Sí	-
10	A.R.	Ocho	Violencia física Violencia psicológica (sospecha de *bullying* en la escuela) Negligencia de protección	Sí	Violencia psicológica (alienación parental)
11	S.G.	Once	Violencia autoprovocada o autoinfligida	Sí	Violencia sexual
12	D.V.	Quince	Violencia autoprovocada Negligencia de protección	Sí	-
13	B.G.	Quince	Trabajo infantil Negligencia de protección, salud y escuela	Sí	-
14	J.C.	Ocho	Violencia sexual Negligencia de protección	Sí	Violencia psicológica
15	L.P.	Siete	Violencia autoprovocada Violencia psicológica Negligencia de protección y salud	Sí	-
16	V.L.	Doce	Trabajo infantil Negligencia estructural y de protección	Sí	-
17	B.G.	Ocho	Violencia sexual Negligencia de protección	Sí	-

Para la etapa de desarrollo del *serious game* se estableció el género simulación, por medio del *software Unity*®, en perspectiva para ambiente 2D, compatible con las plataformas *Android*, iOS y navegador (itch.io).

Aunque el juego no se caracteriza como una simulación clínica tradicional en ambiente presencial, su concepción fue orientada por los principios establecidos en los *Healthcare Simulation Standards of Best Practice*® de la *International Nursing Association for Clinical Simulation and Learning* (INACSL). La estructura del juego integra, de forma adaptada al ambiente digital, las tres fases esenciales de la simulación: *prebriefing*, simulación y *debriefing*.

La fase de *prebriefing* fue operacionalizada por medio de un caso clínico tutorial introductorio, que orienta al jugador respecto al ambiente, a los objetivos y a las funcionalidades del juego. El jugador asume el papel de enfermero o médico responsable por la atención clínica de niños y adolescentes en contexto de APS.

En ese escenario se presentan cajas de diálogo con orientaciones específicas. Este apoyo inicial busca garantizar la comprensión del funcionamiento del sistema y favorecer la adquisición gradual de autonomía en las consultas subsiguientes. En seguida, el juego presenta las informaciones básicas del paciente, tales como nombre, edad, fecha de la consulta e identificación del acompañante. Esas informaciones contribuyen a la contextualización del caso y al inicio del razonamiento clínico.

La interacción se intensifica a partir de la aparición de una caja de diálogo asociada a un ícono de estetoscopio. A partir de ese momento, el jugador observa la interacción entre el profesional y el paciente o acompañante, representados por imágenes. El ícono visible en la interfaz se actualiza conforme el personaje en escena.

La fase de simulación fue compuesta por 16 casos clínicos ficticios, en los cuales durante las interacciones se realiza consulta, anamnesis, examen físico, registra datos clínicos en la historia clínica y toma decisiones basadas en los protocolos de la Red de Protección al Niño y al Adolescente en situación de riesgo. Tales acciones confieren dinamismo al escenario y posibilitan decisiones clínicas fundamentadas.

Entre las opciones disponibles están: señalizar el caso como sospechoso de violencia, mantener esa señalización o efectuar la notificación de sospecha de violencia o del acto concretizado.

La fase de *debriefing* ocurre por medio de *feedbacks* automatizados a lo largo del *gameplay*, destacando aciertos y lagunas relacionados con la notificación obligatoria.

El juego fue titulado *Take Care*. La elección fue justificada por el término en inglés tener el significado literal de “cuidar de algo o alguien”. *Take Car*e se encuentra en proceso de registro de *software*.

En la Figura 2 se presentan ejemplos de pantallas del tutorial del *serious game* desarrollado.

**Figura 2-:** Ejemplo de las pantallas del tutorial del *serious gameTake Care*. Curitiba, PR, Brasil, 2024

Los 48 expertos que realizaron la evaluación del *serious game* estaban en la franja etaria predominante de 40 a 49 años (29%), seguida de 30 a 39 años (28%). Con relación a la formación académica, 48% poseían posgrado, siendo solo una *stricto sensu*, nivel maestría. En cuanto al tiempo de experiencia, se verificó que 50% de ellos tenían más de 21 años de experiencia y solo 18% de los participantes tenían un tiempo entre dos y cinco años.

Todos los expertos relataron afinidad con la tecnología y 75% respondieron que no poseían contacto con juegos digitales.

En relación con la experiencia con educación permanente, 37,5% informaron haber realizado la última participación en la EPS hacía menos de un año; 54% entre uno y 10 años; y 4,5% más de 10 años. Del total de expertos, 33% afirmaron que no habían tenido contacto con el tema de la violencia contra niños y adolescentes en la EPS.

En cuanto a los enfoques utilizados para la EPS sobre el tema de la violencia, el modelo más citado fue la clase expositiva, con ocho participaciones, seguido por la discusión de casos, con siete participaciones. Otros modelos incluyeron: curso de corta duración (cinco participaciones), rueda de conversación (dos participaciones) y conferencia (tres participaciones).

En relación con los resultados del *gameplay*, el tiempo mínimo de ejecución estuvo entre 13 y 15 minutos, mientras que el tiempo máximo registrado se situó entre 21 y 30 minutos. Durante la experiencia simulada, 41% de los participantes identificaron los casos presentados como sospechosos de violencia y 29% realizaron la notificación obligatoria.

El análisis estadístico por la prueba de alfa de Cronbach fue de 0,968. Con base en valores estandarizados, el alfa de Cronbach fue de 0,970, demostrando consistencia casi perfecta. La Tabla 1 presenta la media y la desviación estándar de cada respuesta en todos los dominios evaluados.

**Tabla 1-:** Media y desviación estándar de los ítems de la escala *e-GameFlow*. Curitiba, PR, Brasil, 2024

**Cuestiones**	**Media (n=48)**	**Desviación estándar**
C*		
C1 - ¿El juego capta mi atención?	4,20	0,889
C2 - ¿Presenta contenido que estimula mi atención?	4,27	0,811
C3 - ¿La mayoría de las actividades está relacionada con la tarea del aprendizaje?	4,33	0,801
C4 - ¿Ninguna distracción de la tarea es destacada?	4,00	0,957
C5 - ¿En general, consigo mantenerme concentrado en el juego?	4,39	0,786
C6 - ¿Soy distraído de tareas en las cuales debería concentrarme?	3,18	1,185
C7- ¿Estoy sobrecargado con tareas que parecen sin importancia?	3,20	1,207
C8 - ¿La carga de trabajo del juego es adecuada?	3,98	0,989
H ^†^		
H1 - ¿Disfruto el juego sin aburrirme o ponerme ansioso?	4,18	0,950
H2 - ¿La dificultad es adecuada?	3,90	0,941
H3 - ¿Existen “pistas” que ayudan en la tarea?	4,02	1,010
H4 - ¿Presenta soporte en línea que ayuda en la tarea?	3,76	1,128
H5 - ¿Presenta video o audio que ayudan en la tarea?	3,69	1,211
H6 - ¿Mis habilidades aumentan conforme el juego avanza?	4,14	0,866
H7 - ¿Estoy motivado por la mejora de mis habilidades?	4,12	1,053
H8 - ¿Los desafíos aumentan conforme mis habilidades aumentan?	3,94	0,922
H9 - ¿Presenta nuevos desafíos a un ritmo adecuado?	4,00	0,890
H10 - ¿Presenta diferentes niveles de desafíos que se adaptan a los distintos jugadores?	3,71	1,061
A ^‡^		
A1 - ¿Tengo sensación de control del menú?	3,24	1,164
A2 - ¿Tengo sensación de control sobre funciones y objetos?	3,37	1,035
A3 - ¿Tengo sensación de control sobre las interacciones entre funciones y objetos?	3,45	0,959
A4 - ¿Es posible cometer errores que impiden el avance del juego?	3,04	0,999
A5 - ¿Puedo recuperarme de cualquier error cometido?	2,69	1,103
A6 - ¿Siento que puedo usar cualquier estrategia?	3,04	0,978
A7 - ¿Tengo sensación de control e impacto sobre el juego?	3,57	1,021
A8 - ¿Sé el próximo paso en el juego?	3,51	1,023
A9 - ¿Tengo sensación de control sobre el juego?	3,37	1,035
G ^§^		
G1 - ¿Los objetivos generales son presentados al inicio del juego?	4,20	0,957
G2 - ¿Los objetivos generales son presentados claramente?	4,20	0,979
G3 - ¿Los objetivos intermedios son presentados en el lugar apropiado?	4,06	0,944
G4 - ¿Los objetivos intermedios son presentados claramente?	4,00	0,979
G5 - ¿Entiendo los objetivos del aprendizaje a través del juego?	4,22	0,963
F ^||^		
F1 - ¿Recibo *feedback* de mi progreso en el juego?	4,08	1,017
F2 - ¿Recibo *feedback* inmediato de mis acciones?	3,78	1,177
F3 - ¿Soy notificado sobre nuevas tareas inmediatamente?	4,02	0,968
F4 - ¿Soy notificado sobre nuevos eventos inmediatamente?	3,98	1,010
F5 - ¿Recibo información sobre el éxito o fracaso de objetivos intermedios inmediatamente?	3,51	1,120
F6 - ¿Recibo información sobre mi *status* , como nivel o puntuación?	3,31	1,342
I ^¶^		
I1 - ¿Olvido el tiempo mientras juego?	3,88	1,092
I2 - ¿Olvido las cosas a mi alrededor mientras juego?	3,88	1,033
I3 - ¿Olvido los problemas del día a día mientras juego?	3,76	1,011
I4 - ¿Siento una noción de tiempo alterada?	3,37	0,951
I5 - ¿Puedo mantenerme involucrado con el juego?	4,04	0,999
I6 - ¿Me siento emocionalmente involucrado con el juego?	3,88	0,992
I7 - ¿Me siento visceralmente involucrado con el juego?	3,35	1,217
K ^**^		
K1 - ¿El juego mejora mi conocimiento?	4,29	0,913
K2 - ¿Capto las ideas básicas del contenido presentado?	4,43	0,791
K3 - ¿Intento aplicar el conocimiento en el juego?	4,39	0,812
K4 - ¿El juego motiva al jugador a integrar el contenido presentado?	4,33	0,899
K5 - ¿El juego motiva al jugador a integrar el contenido presentado?	4,33	0,922

*C = Concentración; ^†^H = Desafío; ‡A = Autonomía; §G = Claridad de los objetivos; ^||^F = *Feedback*; ^¶^I = Inmersión; ^**^K = Mejora del conocimiento

Los resultados obtenidos para la escala *eGameFlow* indican que la media de los ítems evaluados fue de 3,831, con valores que variaron entre 2,694 (mínimo) y 4,429 (máximo), resultando en una amplitud de 1,735 y una razón entre el valor máximo y el mínimo de 1,644. La varianza de las medias de los ítems fue de 0,177. Con respecto a la varianza de los ítems, se observó una media de 1,016, con valores que oscilaron entre 0,617 y 1,800, correspondiendo a una amplitud de 1,183 y a una razón entre el valor máximo y el mínimo de 2,916. La varianza de esos valores fue de 0,061.

Todos los participantes afirmaron que jugarían nuevamente, 92% se sintieron seguros al jugar y 89% no pensaron en desistir del juego.

## Discusión

En la elaboración de los casos se utilizaron dos o más tipos diferentes de violencia perpetrada a la misma niña o adolescente. Aun así, los expertos identificaron otro tipo de violencia en algunos casos. Esta superposición intensifica los impactos y está alineada con investigación de autores que señalan que un tipo de violencia no es identificado de forma aislada, predominantemente^(16)^.

Durante la elaboración de los casos se consideró que la violencia física y sexual son las más reconocidas por los profesionales de salud y que ellos tienen dificultad para reconocer otros tipos de violencia. Por lo tanto, al concebir los casos con objetivo de simulación para EPS, la superposición de tipos de violencia reconocidos y tipos más difíciles de identificar, por no presentar señales explícitas^(16)^, fue una estrategia importante.

En relación con los expertos seleccionados en la primera etapa de la investigación, la diversidad de formación profesional contribuyó a mayor amplitud de los casos, al identificar tipos de violencia que posibilitaron la superposición en los casos clínicos. Este resultado está alineado con la dificultad de identificación y la complejidad de los casos evaluados en las Redes de Atención a la Salud^(15)^, lo que refleja la relevancia de la interdisciplinariedad en la construcción del conocimiento y en la práctica en salud.

En la etapa de desarrollo del juego, con propósito de tecnología educacional, después de la elección del tema, además del foco en el aprendizaje es necesaria la creación de una interfaz intuitiva y fácil de navegar, para promover el *engagement* y asegurar que los usuarios consigan acceder fácilmente a los contenidos siempre que lo deseen^(17)^.

Así, en el desarrollo del juego, el uso de casos clínicos ficticios fue esencial para sustentarlo y para definirlo en el género de simulación, que utilizó las mecánicas básicas de jugabilidad, utilizando el *software Unity*®, creando perspectiva para ambiente 2D, compatible con las plataformas *Android*, iOS y navegador (itch.io).

El *Unity*® se configura como el motor de juego ampliamente empleado en la creación de aplicaciones digitales interactivas, con soporte a ambientes bidimensionales (2D). Su arquitectura permite que los proyectos sean ejecutados en diferentes sistemas operativos y dispositivos, como *smartphones*.

El *Unity*® fue utilizado debido a su interfaz gráfica intuitiva y a una extensa base de documentación técnica, factores que favorecen tanto a principiantes como a desarrolladores experimentados en la construcción de experiencias interactivas y gamificadas^(18)^.

Desarrollado con base en los principios de la simulación clínica^(19)^, *Take Care* ofrece al jugador la oportunidad de asumir el papel de enfermero o médico de la APS, en el contexto de atención a niños y adolescentes. Durante la simulación, el jugador interactúa con diferentes perfiles de usuarios y situaciones clínicas, siendo desafiado a ejercer razonamiento clínico y sensibilidad para plantear hipótesis de violencia.

Esta dinámica interactiva promueve la inmersión del jugador en un escenario realista, en el que la identificación de situaciones de riesgo se da a partir de la evaluación clínica cuidadosa, de la interpretación del lenguaje no verbal, del análisis de señales comportamentales y de las posibles inconsistencias en los relatos. También se consideran elementos sutiles como pequeños signos físicos, indicios de negligencia e indicadores psicosociales, todos ellos descritos en la literatura^(16)^.

El desarrollo de habilidades para identificar situaciones de abuso sexual y buscar ayuda fue el resultado identificado en un juego educacional cuyo objetivo y nombre reflejan el concepto de crear un ambiente virtual en el cual niños de ocho a diez años exploran e interactúan de forma segura^(17)^. Del mismo modo, aunque con temática y público objetivo distintos, *Take Care* fue desarrollado en ambiente virtual con escenarios y atenciones realistas, en los cuales médicos y enfermeros pueden explorar e interactuar con contenido sensible de forma segura. Esta propuesta busca favorecer el desarrollo del razonamiento clínico, de la sensibilidad profesional y de la capacidad de identificar y notificar situaciones de violencia contra niños y adolescentes.

Esta concepción dialoga con el potencial de los *serious games* para simular situaciones clínicas reales, proporcionando a los usuarios experiencias inmersivas que favorecen el aprendizaje activo y el perfeccionamiento de habilidades clínicas.

Un ejemplo es el juego *Virtual ER*, una sala de emergencia virtual orientada a la educación interprofesional de estudiantes de medicina y enfermería^(19)^. Esta herramienta utiliza una plataforma digital para simular atenciones en salas de emergencia, promoviendo el trabajo en equipo y la toma de decisiones clínicas en un ambiente seguro y controlado^(19)^.

De forma complementaria, el juego *Take Care* presenta una propuesta innovadora al transponer la simulación clínica para enfermeros y médicos en el contexto de APS, con foco específico en la identificación y notificación de la violencia contra niños y adolescentes.

Al insertar al jugador en escenarios realistas y basados en casos clínicos ficticios, *Take Care* puede ampliar el alcance de actuación de los profesionales de salud, estimulando el razonamiento clínico para el reconocimiento de señales subjetivas de sufrimiento, muchas veces no identificadas en la práctica asistencial.

Aunque no identificado de forma prevalente en los resultados, se espera que el uso de *Take Care* estimule la comprensión sobre la importancia de la notificación de los casos de violencia y de la articulación con los mecanismos de la Red de Protección, promoviendo la integración entre habilidades clínicas, sensibilidad ética y actuación intersectorial.

En relación con la predominancia de enfermeros en la evaluación del *serious game*, esta es reflejo de la presencia de este profesional en la asistencia en APS. Este punto es destacado en estudio que analizó el perfil y las prácticas de enfermeros de la APS en la región Sur de Brasil, que evidenció la centralidad de enfermeros en la prestación del cuidado directo a la población, reforzando su predominancia frente a otros profesionales de salud^(19)^. Sin embargo, para la evaluación del juego, también fue importante la participación de médicos, considerando que ellos realizan atención directa a niños y adolescentes en la APS y son profesionales que identifican situaciones de violencia durante las consultas.

Otro punto para destacar es la madurez profesional de los expertos incluidos en la evaluación de *Take Care*. La madurez también fue relatada en estudio brasileño, que sugirió un perfil de expertos con trayectoria consolidada y experiencia acumulada en el cuidado en salud^(20)^. Tal perfil puede favorecer una evaluación más criteriosa determinada por las distintas fases generacionales.

El uso de criterios para selección de expertos para evaluar tecnologías educacionales no posee consenso, pero convergen en la necesidad de aspectos relacionados con la formación académica y actuación/experiencia profesional^(21)^. Así, se sugiere que en estudios futuros uno de los criterios de elección para evaluación de tecnologías educacionales para el cuidado en salud sea fundamentado por la madurez profesional.

Los resultados referentes a la proximidad de los expertos con tecnologías reflejan, en parte, el hecho de que la sociedad ha pasado por una transformación social significativa, alterando profundamente las formas de pensar, comunicarse, relacionarse y trabajar. Estos cambios son impulsados por la revolución digital^(22)^.

Era esperado que los expertos demostrasen afinidad con el uso de tecnologías, dado el crecimiento del uso de recursos digitales en la salud. Sin embargo, los datos de este estudio indicaron que esa afinidad no se extendió a los juegos digitales. La escasa divulgación entre profesionales de la salud sobre *serious games* y sus aplicaciones educativas puede haber limitado el acceso previo a esa experiencia.

A pesar de ello, la ausencia de familiaridad no comprometió la interacción con el *serious game* ni la comprensión de los contenidos propuestos. Eso refuerza el potencial inclusivo de la tecnología, incluso entre profesionales con diferentes niveles de exposición a recursos lúdico-digitales.

También es importante destacar el escenario de baja regularidad o de acceso a la EPS. Tal escenario puede sugerir fragilidad de la institucionalización de la EPS en los servicios de salud e indicar lagunas de actualización que comprometen la calificación del cuidado. A su vez, es necesario analizar con parsimonia, pues la efectividad de la EPS es frecuentemente limitada por factores como la sobrecarga de trabajo, falta de inversión y presencia de un modelo biomédico que privilegia metas e indicadores cuantitativos de asistencia^(23)^.

En cuanto al *gameplay*, primeramente es necesario destacar que el tiempo de juego fue efectuado predominantemente entre 13 y 15 minutos. El tiempo puede haber sido reflejo de la planificación adecuada, en que *Take Care* fue proyectado para mantener la atención y alcanzar los objetivos sin sobrecargar a los jugadores.

En el transcurso del *gameplay*, la decisión de marcar un caso como sospecha o confirmación de violencia se atribuye a la decisión clínica del profesional. Comprendiendo que la notificación es una obligación de todos los profesionales de salud, constituyéndose como etapa crucial para el accionamiento de la red intersectorial de protección^(24)^, es preocupante el hallazgo de que jugadores no indicaron la notificación al final del caso.

Estos hallazgos dialogan con la literatura nacional e internacional^(25)^, que señalan dificultades de profesionales de salud para la identificación de situaciones de violencia contra niños y adolescentes. Tales lagunas implican no solo la identificación de las señales, especialmente las más sutiles o invisibles, sino también la toma de decisión frente a la notificación obligatoria^(24)^.

En cuanto a la evaluación del juego por medio de la escala *eGameFlow*, los resultados indicaron desempeño satisfactorio en la mayoría de los dominios analizados. Los resultados del dominio “Mejora del conocimiento” sugieren que *Take Care* favoreció la comprensión, la integración y el interés por el contenido, reforzando su potencial como herramienta de apoyo a la EPS.

De modo similar, los dominios “Concentración” y “Claridad de los objetivos” también presentaron resultados positivos, con destaque para ítems como “consigo mantenerme concentrado en el juego” y “entiendo los objetivos del aprendizaje”. Esos hallazgos sugieren que el diseño del juego tuvo éxito en mantener el foco del jugador y presentar metas de forma clara, favoreciendo el *engagement* cognitivo durante la simulación interactiva.

Se encuentran en la literatura investigaciones sobre análisis y evaluación de *serious game* semejantes a los resultados presentados en este artículo. Investigación que analizó *serious game* en el área de salud para el diseño y la evaluación de juegos serios orientados a la educación en salud, involucrando profesionales de salud, pacientes y público en general, describió que 58% de los juegos buscaban perfeccionar el conocimiento y 41% se enfocaban en el desarrollo de habilidades.

Otro estudio sobre el uso de *serious game* que evaluó la tecnología y la efectividad de la acción educativa en la EPS evidenció mejora del conocimiento después de la estrategia^(26)^. En ambos la evaluación del *serious game* fue positiva en el dominio de mejora del conocimiento, con media por encima de 4,00 en todos los ítems evaluados, resultado corroborado por los resultados de la presente investigación, lo que sugiere la finalidad educativa de un *serious game*.

Los participantes demostraron receptividad positiva a *Take Care*, cuando afirmaron que jugarían nuevamente, se sintieron seguros al jugar y no desistirían. Eso sugiere que el juego permitió una experiencia inmersiva.

Un estudio que desarrolló *serious game* en el cual el público objetivo eran niños y lo evaluó con la misma herramienta utilizada en la presente investigación presentó resultados semejantes en relación con la categoría autonomía; en el estudio se optó por ajustar la interfaz en la versión final del juego, indicando el ítem siguiente y permitiendo que el jugador se preparase adecuadamente para la próxima etapa^(17)^. Esa estrategia será utilizada para ajustes en *Take Care*.

Este estudio contribuye al avance del conocimiento científico al presentar una propuesta innovadora de tecnología educacional orientada a la identificación y notificación de la violencia contra niños y adolescentes en la APS. El *serious game Take Care* demostró potencial para fortalecer políticas públicas de protección, siendo herramienta para auxiliar en la reducción de la invisibilidad de ese fenómeno.

Además, *Take Care* se configura como una estrategia promisoria para prácticas de cuidado integrales y humanizadas, al promover la sensibilización de los profesionales para señales y síntomas invisibles de tipologías de violencia, para los cuales ellos no tomarían decisión de notificar. De forma complementaria, *Take Care* puede ser utilizado en cursos del área de la salud.

Entre las limitaciones de la investigación están: (i) el tiempo exigido para el desarrollo del juego, que es prolongado y exige adecuaciones de recorrido que, a veces, no son adecuadas a la temporalidad del recorrido académico del *stricto sensu*; (ii) la dificultad en mantener el *engagement* continuo de los equipos interdisciplinarios involucrados en las diferentes etapas del proyecto; y (iii) el escenario de investigación darse en una capital con histórico consolidado de actuación en Red de Protección, lo que puede limitar la generalidad de los resultados y necesitar ajustes en la herramienta o inserción de nuevos casos clínicos con características distintas.

## Conclusión

Los conceptos que fundamentan los tipos de violencia, las directrices del Protocolo de la Red de Protección al Niño y al Adolescente en situación de riesgo y el uso de un guion instruccional fueron adecuados y son esenciales para la elaboración de casos clínicos que representan fenómenos de la realidad.

El *serious game Take Care* fue desarrollado, por equipo interdisciplinario, en el género de simulación, en el *software Unity*®, en perspectiva para ambiente 2D, compatible con las plataformas *Android*, iOS y navegador (itch.io), lo que le permite fácil acceso.

La evaluación de *Take Care* alcanzó alfa de Cronbach 0,970. El juego presentó desempeño satisfactorio en la evaluación de satisfacción, con media de los ítems evaluados de 3,831 (mínimo de 2,694 y máximo de 4,429), con amplitud de 1,735 y razón entre el valor máximo y el mínimo de 1,644. Los dominios de mayor satisfacción fueron “Mejora del conocimiento”, “Concentración” y “Claridad de los objetivos”.

Se espera que el *serious game Take Care* contribuya con enfermeros y médicos de la APS para identificación y notificación de situaciones de violencia, reconociendo el fenómeno como una expresión compleja y multifacética, enraizada en las relaciones sociales.

## Data Availability

El conjunto de datos de este artículo está disponible en la tesis de doctorado ““Casos clínicos e jogo sério para a educação permanente em saúde para identificação de violência a criança e adolescente”“, en el enlace https://pergamum-biblioteca.pucpr.br/acervo/375395.
